# Cubosomes from hierarchical self-assembly of poly(ionic liquid) block copolymers

**DOI:** 10.1038/ncomms14057

**Published:** 2017-01-16

**Authors:** Hongkun He, Khosrow Rahimi, Mingjiang Zhong, Ahmed Mourran, David R. Luebke, Hunaid B. Nulwala, Martin Möller, Krzysztof Matyjaszewski

**Affiliations:** 1 Center for Macromolecular Engineering, Department of Chemistry, Carnegie Mellon University, 4400 Fifth Avenue, Pittsburgh, Pennsylvania 15213, USA; 2 DWI-Leibniz Institute for Interactive Materials, Forckenbeckstr. 50, Aachen 52074, Germany; 3 Department of Chemical and Environmental Engineering, Yale University, New Haven, Connecticut 06511, USA; 4 National Energy Technology Laboratory, United States Department of Energy, P.O. Box 10940, Pittsburgh, Pennsylvania 15236, USA

## Abstract

Cubosomes are micro- and nanoparticles with a bicontinuous cubic two-phase structure, reported for the self-assembly of low molecular weight surfactants, for example, lipids, but rarely formed by polymers. These objects are characterized by a maximum continuous interface and high interface to volume ratio, which makes them promising candidates for efficient adsorbents and host-guest applications. Here we demonstrate self-assembly to nanoscale cuboidal particles with a bicontinuous cubic structure by amphiphilic poly(ionic liquid) diblock copolymers, poly(acrylic acid)-*block*-poly(4-vinylbenzyl)-3-butyl imidazolium bis(trifluoromethylsulfonyl)imide, in a mixture of tetrahydrofuran and water under optimized conditions. Structure determining parameters include polymer composition and concentration, temperature, and the variation of the solvent mixture. The formation of the cubosomes can be explained by the hierarchical interactions of the constituent components. The lattice structure of the block copolymers can be transferred to the shape of the particle as it is common for atomic and molecular faceted crystals.

## Abstract (web summary)

Generally cubosomes are formed by the self-assembly of surfactants such as lipids and are used as adsorbents or in host-guest applications. Here the authors have shown that an amphiphilic block copolymer can form nanoscale cuboidal particles with a bicontinuous cubic phase.

Nature creates biological systems with a significant level of complexity, whereas it relies on a limited number of building units, such as amino acids, nucleotides and lipids. Structural variation and function exploits self-assembly into complex supramolecular architectures mediated by weak, noncovalent bonds (hydrogen bond, ionic bond, hydrophobic interaction, van der Waals interaction, and so on) in a delicate and synergistic manner^1^. Even though the biological self-assembly is extremely intricate, scientists have been striving for unveiling and utilizing these principles for the design of functional materials. Block copolymer (BCP) self-assembly is one of the most prominent examples of this approach. Self-assembly of BCPs into discrete nanoscale objects possessing controlled structures and tailored functionalities is of considerable interest for applications in many fields, such as biomedicine, stimuli-responsive materials, controlled delivery systems, membranes or catalysts^2^,^3^,^4^,^5^,^6^. Driven by minimization of energetically unfavourable segment/solvent interactions, amphiphilic BCPs aggregate in selective solvents to form a wide variety of morphologies, such as spheres, cylinders, vesicles (polymersomes), ribbons, films, fibres, tubules and multigeometry nanoparticles^7^,^8^,^9^,^10^,^11^,^12^,^13^,^14^.

Within these morphologies bicontinuous mesostructures form a special subgroup characterized by their 3D percolating phase structure. Although bicontinuous phase structures are well known, the range of their stability is limited and dispersed synthetic particles with bicontinuous cubic liquid crystalline nanostructures, so called cubosomes, are mostly known only for low molecular weight surfactants^15^,^16^,^17^,^18^. On the other side, cubic-shaped bio-tissues are critical elements of some biological processes, for example, cuboidal epithelia cells^19^ and cubic phases in bacterial cell membranes^20^,^21^.

Cubosomes are typically formed by low molecular weight surfactants (most commonly, glycerol monooleate) in water, and often in the presence of stabilizers^22^,^23^,^24^,^25^. These cubosomes consist of two continuous but non-intersecting hydrophilic regions separated by a surfactant bilayer, which is arranged in a thermodynamically favourable periodic 3D structure by contorting the bilayer into the shape of infinite periodic minimal surfaces with zero mean curvature^26^,^27^. The special properties of cubosomes, such as high internal surface area, internal accessibility for hydrophilic, hydrophobic and amphiphilic molecules, enable their applications in food science and health-care products, controlled delivery vehicles, and as templates for materials synthesis^15^,^22^,^28^,^29^,^30^,^31^,^32^. The fact that cubosomes consisting of a BCP are scarce is most likely due to the narrow range of assembling conditions required for bicontinuous separation. For BCPs, formation of a bicontinuous morphology is known to alleviate the entropic penalty associated with polymer segment stretching when the polymer are forced to form a brush like arrangement across the interface^33^. Furthermore, it should be noted that the bicontinuous cubosome structure might be mostly metastable, leading to their formation under kinetic control^34^,^35^,^36^.

Here we present cuboidal-shaped cubosomes with an internal bicontinuous cubic phase that were formed by self-assembly of poly(ionic liquid) block copolymers (PIL-BCPs). Remarkably, the cubic internal structure has been transformed to the overall shape of the particle as it is well known for atomic and molecular faceted crystals, but so far never observed for BCPs. Poly(ionic liquid)s (PILs) are a special type of polyelectrolytes which carry an ionic liquid moiety on each repeating unit^37^,^38^,^39^,^40^,^41^. So far, there have been only a few reports on the self-assembly of PILs in solutions, such as vesicles or micelles formed by random copolymers or BCPs of PILs (refs 42, 43, 44), and nanoparticles with highly ordered concentric multilamellar or unilamellar vesicular inner structures formed by PILs with quaternary ammonium side chains^37^,^45^. Our work demonstrates that PIL-BCPs can form cuboid particles with an internal bicontinuous morphology. The specific self-assembling conditions indicate that the particles formation is controlled by hierarchical interactions. It is envisioned that such system will provide improved chemical and mechanical stability. The unique structures and properties of the PIL-BCPs cubosomes may provide further understanding of bicontinuous self-assembly in biological systems and biomimetic chemistry^46^,^47^,^48^,^49^.

## Results

### Synthesis and characterizations of PIL-BCPs

**Figure 1:**
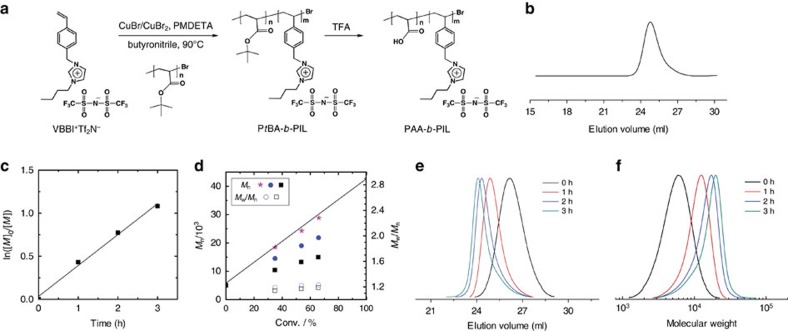
Block copolymer synthesis and characterization. (**a**) Synthetic approach to PAA-*b*-PIL through ATRP using P*t*BA-Br as the macroinitiator. (**b**) GPC elution of P*t*BA_45_-*b*-PIL_23_ in THF containing 10 mM LiTf_2_N and 10 mM 1-butylimidazole. (**c**) Plot of ln([*M*]_0_/[*M*]) versus time. (**d**) Plot of *M*_n_ and *M*_w_/*M*_n_ versus conversion. *M*_n_ was accessed by NMR (pink stars). *M*_w_ was obtained from GPC analysis using linear polystyrene (black squares) and polyVBBI^+^Tf_2_N^−^_RAFT_ standards (blue circles). Samples were taken at different times from the polymerization. (**e**,**f**) GPC traces of polyVBBI^+^Tf_2_N^−^ (in THF containing 10 mM LiTf_2_N and 10 mM 1-butylimidazole) calibrated using linear polystyrene standards. Conditions: [VBBI^+^Tf_2_N^−^]_0_/[P*t*BA-Br (*M*_n_=5950)]_0_/[CuBr]_0_/[CuBr_2_]_0_/[PMDETA]_0_=70/1/1.9/0.1/2, VBBI^+^Tf_2_N^−^/butyronitrile=1/1 (w/w), 90 °C.

The synthesis of well-defined PIL-BCPs was achieved by atom transfer radical polymerization (ATRP) (refs 50, 51, 52) of ionic liquid monomers. As shown in Fig. 1a, poly(*tert*-butyl acrylate) (P*t*BA) synthesized by activators regenerated by electron transfer (ARGET) ATRP (ref. 53) was used as the macroinitiator for the synthesis P*t*BA-*b*-PIL via ATRP using IL monomer of 1-(4-vinylbenzyl)-3-butyl imidazolium bis(trifluoromethylsulfonyl)imide (VBBI^+^Tf_2_N^−^). First-order kinetics was observed for the conversion of the monomer measured by means of nuclear magnetic resonance (NMR) spectroscopy (Fig. 1c). The number averaged molecular weights (*M*_n_) values obtained by NMR analysis correlated very well with the theoretical values of *M*_n_ (Fig. 1d).

PIL-BCPs with varying chain lengths of both blocks were synthesized with precisely controlled degrees of polymerization of each block (Supplementary Figs 1–10 and Supplementary Table 1). Gel permeation chromatography (GPC) analysis of PIL-BCPs was performed to confirm the control of the polymerization by monitoring the evolution of molecular weights and molecular weight distribution. Tetrahydrofuran (THF) containing LiTf_2_N and 1-butylimidazole was used as the eluent; linear polystyrene and self-made PILs were employed as the calibration standards^54^. The GPC traces of P*t*BA-*b*-PIL progressively shifted to higher molecular weight with polymerization time (Fig. 1e,f). The resulting PIL-BCPs typically displayed a monomodal molecular weight distribution and low dispersity *Ð*=*M*_w_/*M*_n_ ∼1.1-1.3. The *tert*-butyl groups of P*t*BA-*b*-PIL were cleaved by trifluoroacetic acid (TFA) to yield PAA-*b*-PIL (Supplementary Fig. 11).

### Morphology of the cubosomes from self-assembly of PIL-BCPs

**Figure 2:**
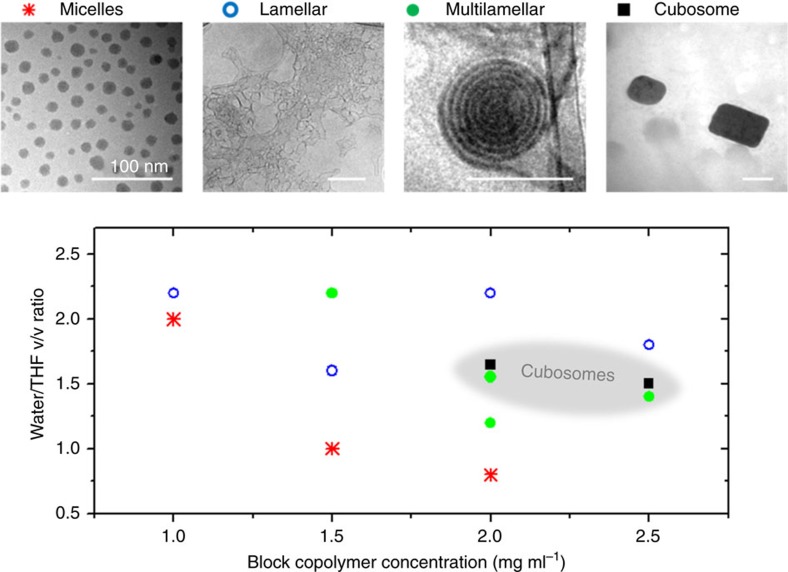
Morphologies of self-assembled particles. Morphology formation of PAA_45_-*b*-PIL_23_ in water/THF mixtures as a function of block copolymer concentration and solvent composition. Four regions of interest were observed—micelles, lamellae, multilamellar vesicles and cubic particles. The regions with overlapped colours are metastable. The scale bar in TEM micrographs is 100 nm.

A systematic exploration of the variation of self-assembled structures of PAA_45_-*b*-PIL_23_ as a function of the solvent composition and BCP concentration is shown in Fig. 2. Here the index numbers represent the number average of the degree of polymerization of the respective block. The self-assembly experiment was conducted by dissolving PAA_45_-*b*-PIL_23_ in THF and then adding water. The PAA block was soluble in both THF and water, while PIL block was only soluble in THF. Structures were analysed after stirring for 2 days. Four different types of particles (that is, micelles, lamellae, multilamellar vesicles and cubic particles) were observed by cryogenic transmission electron microscopy (cryo-TEM) and the representative electron micrographs of the morphologies are shown in Fig. 2. To which extent each morphology was formed is governed by the BCP concentration and water/THF volume ratio (*R*). The diagram pinpoints a relatively narrow regime of cubic particles. Only spherical particles were observed when *R*=1.2, 1.4 or 1.8, while some cuboidal particles were formed in addition to the spherical particles when *R=*1.5 or 1.6 (with the highest yield of cuboidal particles at *R*=1.6) (Fig. 2 and Supplementary Fig. 12).

It is well established that the most stable structure is defined by the bending energy, close packing entropy and solubility parameter (interaction between blocks and solvents)^55^. The morphology formation of PAA_45_-*b*-PIL_23_ in water/THF mixtures, however, demonstrates coexistence of different morphologies over rather large range of conditions. Generally, coexistence of different morphologies is often observed in BCP self-assembly in solution caused by the effect of dispersity in block lengths of BCPs. Furthermore, coexistence can be expected if the structure formation is kinetically controlled, for example, by vitrification of the aggregates of different morphologies during water addition^56^,^57^. Additionally, nucleation and growth rates can control the formation of metastable structures. In particular for amphiphilic systems like BCPs, inflated local concentration favour the stabilization of nuclei and formation of structures, which could otherwise be found only in highly concentrated solutions^55^.

**Figure 3:**
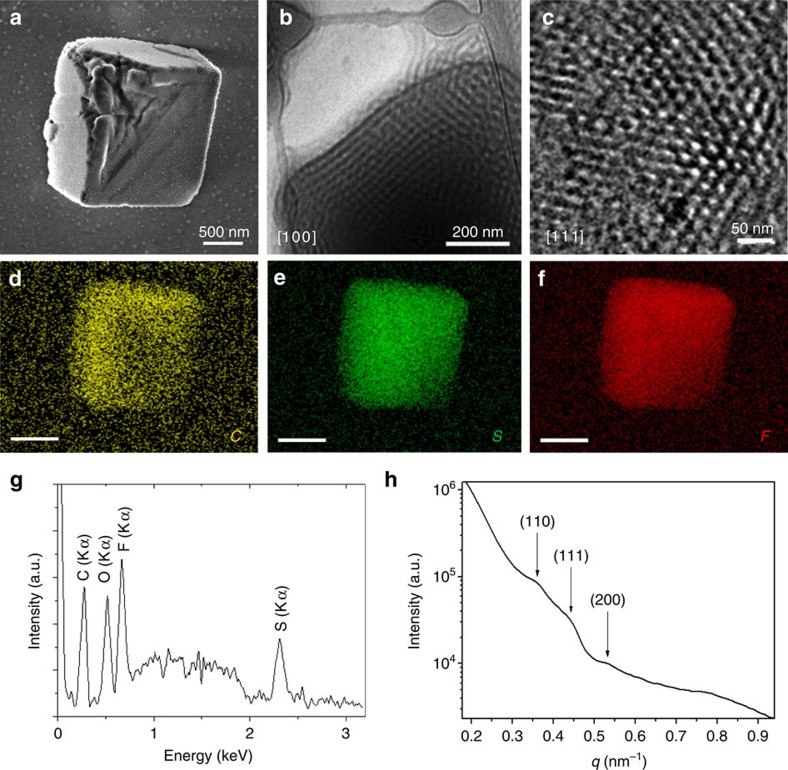
Triply periodic structures of cubosomes. The electron micrographs of the self-assembled aggregates of PAA_45_-*b*-PIL_23_ in water/THF solution recorded 2 days after adding water and aging the dispersion at 21 °C. Self-assembling conditions: 2 mg ml^−1^ THF solution of PAA_45_-*b*-PIL_23_, water/THF volume ratio (*R)=*1.6. (**a**) SEM micrograph of the dried cubosomes after dialysis. (**b**) Cryo-TEM image of the [100] facet of a cuboid particle. (**c**) Enlarged surface image recorded in the central area of a cuboid particle from the [111] facet. (**d**–**f**) Elemental mappings (scale bar: 100 nm) show the uniform distribution of the elements C, S and F throughout the cuboid structure. The homogeneous distribution is in agreement with the interpenetrating bicontinuous structure expected for cubosomes. (**g**) Energy-dispersive X-ray spectrum. A Si peak was subtracted that originated from the silicon wafer on which the cubosomes were deposited. (**h**) SAXS diagram indicates a double diamond (*Pn*3*m*) lattice with a=23.5 nm (*q*/q* ∼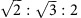).

It should be noted that cuboid shapes are predominantly observed for larger particles while the smaller ones are mostly spherical. As the shape is in the first instance controlled by the balance of the internal packing and the surface energy, formation of the cuboid particles is preferred for those with a smaller surface to volume ratio. The onion-like vesicles consisted of centric multilamellar vesicular structures with layers separating of *ca.* 13 nm (Fig. 2). The 3D shape of cuboidal particles and internal bicontinuous minimal surfaces could be directly observed in the cuboid particles by means of scanning electron microscopy (SEM), TEM and cryo-TEM (Fig. 3a–c, Supplementary Figs 12–16 and Supplementary Movie 1). The cubic particles are truncated at the edges (radius of curvature of 70±15 nm) and consisted of an ordered bicontinuous network of intertwined carboxylic and salt-rich regions with a periodicity of *ca.* 23 nm obtained by TEM micrographs. The dark areas in the cryo-TEM images can be assigned to the PIL blocks containing ion pairs of the imidazolium cations and Tf_2_N^−^ anions, resulting in a higher electron density compared to the PAA blocks. Furthermore, energy-dispersive X-ray spectroscopy analysis showed the uniform distribution of ionic liquid moieties in the cubosomes (Fig. 3d–g).

The internal order of the polymer cubosomes of Fig. 3a was also studied using small-angle X-ray scattering (SAXS). The SAXS results of the polymer cubosomes in water showed a set of very weak peaks that can be attributed to the double diamond (*Pn*3*m*) symmetry (lattice parameter (*a*)=23.5 nm, Fig. 3h). In order to get a better insight into the structure of particles, we also determined the crystallographic indices of the facets of the cubosomes. The cryo-TEM images of cubosome particles (Fig. 3b,c) closely resemble the crystallographic 2D projection of double diamond structure, showing that the particles are single domain^58^. The bicontinuous domain morphology is particularly remarkable for such single domain particles made from BCPs.

A series of control experiments was conducted to study the self-assembly of PAA_45_-*b*-PIL_23_ under varying conditions, including pH, ionic strength, polymer concentration, the addition rate of water and common solvent, and polymer composition. When an aqueous solution of NaOH (pH=9), HCl (pH=1), or NaCl (0.1 M) was added to the THF solution of PAA_45_-*b*-PIL_23_, we observed no cuboidal assembly (Supplementary Fig. 17). This is likely due to the fact that the interchain electrostatic interactions change by the neutralization and electrostatic screening of the PAA segments^59^. When the initial concentrations of PAA_45_-*b*-PIL_23_ THF solutions were 2 or 5 mg ml^−1^, cuboidal assembly was observed; however, when the concentrations were 20, 10, 1 or 0.5 mg ml^−1^, no cuboidal assembly was observed (Supplementary Fig. 18). This might be due to the concentration dependence of nucleation^2^,^56^. Cuboidal assembly resulted at an appropriate rate of addition of water (1.6 ml in 20 s), while no cuboidal assembly was observed at very fast (1.6 ml in 2 s) or very slow (1.6 ml in 30 min) addition rate of water (Supplementary Fig. 19). The sensitivity of the assembly confirms that the process is, at least partially, kinetically controlled^56^. Adding additional THF to the cubosome dispersion resulted in loss of cubosome structures and very few micelles can be observed (Supplementary Fig. 20). Additionally, the nature of the common solvent played a significant role in the formation of the cubosomes. This is expected because the common solvents with different solubility parameters and dielectric constants could directly affect the dimensions of both hydrophilic and hydrophobic domains of the aggregates^60^,^61^. When *N,N*-dimethylformamide was used as the common solvent instead of THF, no cuboidal assembly was observed under similar conditions (Supplementary Fig. 21). Furthermore, the cuboidal assembly was observed for the BCPs having *n*_*IL*_/*n*_*AA*_∼0.5 (that is, PAA_23_-*b*-PIL_12_, PAA_45_-*b*-PIL_23_ and PAA_90_-*b*-PIL_46_), where *n* denotes the repeating unit of each block, but no cuboidal assembly was observed for PAA_23_-*b*-PIL_23_, PAA_45_-*b*-PIL_12_, and PAA_45_-*b*-PIL_98_, PAA_90_-*b*-PIL_23_ (Supplementary Figs 22 and 23).

### Structure evolution as a function of time

**Figure 4:**
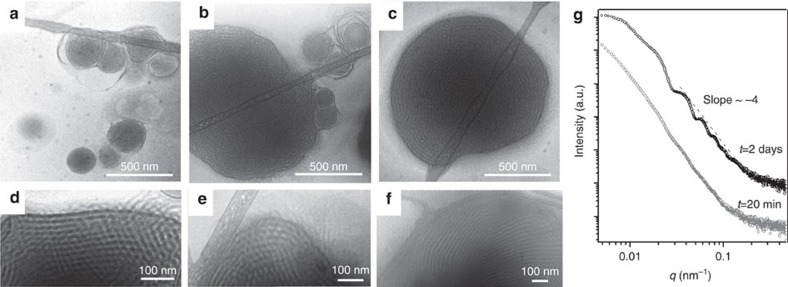
Structure evolution. Cryo-TEM images of the self-assembled aggregates of PAA_45_-*b*-PIL_23_ recorded after 1 h (**a**), 1 day (**b**) and 2 days (**c**) of adding water to the THF solution. Cryo-TEM images of the self-assembled aggregates of PAA_45_-*b*-PIL_23_ aged at 21 °C (**d**), 50 °C (**e**) and 80 °C (**f**). Self-assembling conditions: 2 mg ml^−1^ THF solution of PAA_45_-*b*-PIL_23_, water/THF volume ratio (*R)=*1.6. (**g**) SAXS pattern of PAA_45_-*b*-PIL_23_ solution measured after 20 min and 2 days after adding water.

In order to provide more evidence of the structure evolution, we monitored the self-assembly process of the PIL-BCPs by cryo-TEM micrographs recorded as a function of time. The structure evolution is illustrated by a sequence of representative micrographs in Fig. 4a–c. After the addition of water to the THF solution of PAA_45_-*b*-PIL_23_, the microstructures changed over a 2-day period. The cryo-TEM micrographs recorded after 1 min of adding water (Supplementary Fig. 24) showed the polymers formed irregular aggregates, which then self-assembled and transferred predominantly into multilamellar vesicles within 1 h (Fig. 4a). At this time not all of the vesicles were spherical, but some appeared to be facetted, and cubosomes evolved. The size of the vesicles was much smaller than that of the cubosomes. After 1 day, the number density of the vesicles was decreased and the remaining vesicles were mainly observed in close contact with cubosomes (Fig. 4b). After 2 days, cubosomes with sharp edges appeared (Fig. 4c).

The SAXS data for the PAA_45_-*b*-PIL_23_ structures are shown in Fig. 4g for the shortest (20 min) and the longest (2 days) time intervals between the addition of water and imaging. At *t*=20 min, Porod scattering with *I*(*q*)∝*q*^−4^ indicates many irregular structures with inhomogeneous sizes. This scattering pattern is characteristic for scattering of a phase separated structure with sharp interface. At *t*=2 days, an additional non-oscillating contribution with the slope of *q*^−2.5^ was found at low *q*, indicating the presence of a large amount of truncated cubic particles^62^. This is in agreement with the TEM images showing that the volume ratio of cubosomes to micelles increases to as high as 10^2^ after two days. Moreover, the presence of Porod oscillation at *t*=2 days indicated that the particle size distribution became narrower by time^63^.

The temperature dependence of the aging was investigated with cryo-TEM. As shown in Fig. 4d,e when the sample was aged at 50 °C, the average periodicity was *ca.* 25 nm, which was larger compared to samples aged at 21 °C (*ca.* 23.5 nm). This can be attributed to increased uptake of water in the cubic domains at higher temperatures. Further raising the temperature to 80 °C led to an order-disorder phase transition and no ordered lamellar or bicontinuous structures were observed in this sample (Fig. 4f). The phase transition was likely induced by the elevated temperature that could improve the chain mobility, reduce the incompatibility of the two blocks, break the hydrogen bonds and reduce the number of water molecules hydrating the PIL-BCPs (refs 64, 65). It was also noted that the cubosomes were quite stable at room temperature and they preserved their internal structure even after drying without any need for using stabilizers (Fig. 3a).

## Discussion

The self-assembly process and the resulting aggregate morphologies of the PIL-BCPs in solution are governed by a complex set of factors, such as the nature of the solvents, the temperature, the structure of the BCPs (molecular weight, volume fraction of each block, architecture and the effective interaction energy between blocks), and the processing conditions^7^. The controlled addition of water to a THF solution of PAA-*b*-PIL generated conditions where the hydrophobic PIL block became insoluble while the PAA block was still soluble, and the polymers started to aggregate to form centric multilamellar structures. The lamellar particles had low degree of long range order, which might be attributed to the strong segregation between the ionic and nonionic blocks^66^. On the other hand, the bulky asymmetric structure and charge delocalization of the imidazolium ring and non-coordinating Tf_2_N^−^ led to large anion-cation distance, weak electrostatic interaction, and thus the short range anion-cation ordering was expected to be low^67^. As a consequence, the time needed for the polymers to organize into morphologies in which cations and anions pack more efficiently increased. If BCP chains are forced to quickly assemble into the aggregates by fast addition of excess water, they could do so at the expense of lower perfection of ordering. This was confirmed by the cryo-TEM images in Supplementary Fig. 25 showing organization of polymer chains into irregular lamellar structures (*R*=2.2).

**Figure 5:**
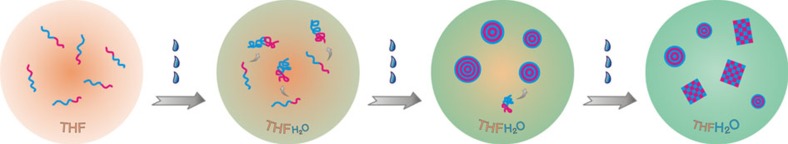
Proposed self-assembling pathway. Schematic illustration of the self-assembly process of PAA-*b*-PIL in THF/H_2_O mixture with the addition of water.

Based on these considerations and the experimental data we have collected so far, we proposed the cubosome formation process schematically depicted in Fig. 5. At the early stage of water addition, the PIL-BCP chains aggregated in a disordered way and the aggregates grew when more water was added. At this stage, the polymer solution unavoidably yields aggregates of differing degrees of order. As the aggregates grew, microphase separation of the two immiscible blocks proceeded, providing a geometric confinement for THF swollen domains^65^. At the same time, higher water contents in the solution caused extraction of THF from the domains and the incompatibility of the two blocks was gradually increased. Now, local rearrangements in the PIL domains were expected to allow more dense and efficient packing of anions and cations, causing a transition from the lamellar phase to the bicontinuous phase to minimize the interface free energy^68^. However, the organization rate in the experiments described here was slow and reorganization could take place even after complete addition of water (*R*=1.6, Fig. 4a–c), leading to an improvement in the overall order of aggregates by transformation from centric vesicular structures to cubosomes. The fact that no cubosomes were observed at low water/THF ratio (*R*=1.2) suggested that the THF fraction in the pre-formed vesicles was still too high for the formation of the bicontinuous cubic phase. The proposed self-assembling mechanism involves a hierarchy of formation steps starting from aggregation, to domain formation controlled by swelling with THF and nucleation controlled morphology transformation upon THF extraction. It combines microphase separation, nucleation and growth of the polymer chains into a united dynamic self-organized precipitation process^65^.

The unique external cuboidal morphology and the internal bicontinuous cubic phase of the cubosomes formed by the PIL-BCPs aggregation might originate from their unique structures and properties. The chemical structures of the PAA-*b*-PIL allow the presence of multiple interactions in the self-assembling process, including hydrogen bonds, ionic bonds (electrostatic interactions), hydrophobic interactions and van der Waals interactions. The hydrogen bonding between carboxyl group and the imidazolium ring play an important role in the self-assembly process, as revealed in some previously reported cases^69^,^70^,^71^,^72^,^73^,^74^,^75^. The interionic interaction of the pendant ionic groups has been shown to be one of the major parameters for the generation of cubic liquid crystalline structure^76^. The PAA_45_-*b*-PS_25_ control sample, an analogue of PAA_45_-*b*-PIL_23_ without ionic groups, did not form cubosomes (Supplementary Figs 26 and 27), indicating the indispensability of the ionic groups. Additionally, a coordination complex could form between the imidazolium cation in the PIL block and the oxygen atom in the carbonyl group of PAA block, causing intra- and inter-chain crosslinking^77^.

Different types of interactions with varying intensities interplay with each other to create a complex environment for self-assembling, providing multidimensional driven forces for the movement and arrangement of molecules. In PAA-*b*-PIL, the multiple intra- and inter-molecular interactions may be unique for the generation of the cuboidal cubosomes, which have not been formed by other BCPs that have lower dimensions of interactions. The self-assembly by hierarchical driven forces could be used for generating structures that are difficult or impossible to form under single or only a few types of self-assembling driven forces. A few examples have been reported on self-assembly processes through hierarchically driven forces that resulted in extraordinary phenomena, including the unique nanoscale square patterns formed by combining supramolecular assembly of hydrogen-bonding units with controlled phase separation of BCPs (refs 73, 78), and the macroscopic molecular self-assembly of an amphiphilic hyperbranched multi-arm copolymer induced by microphase separation and further driven by the hydrogen bonds^74^. The ionic liquid structure provides a means to enlarge the toolbox of hierarchical interactions to be used for the creation of unique self-assembled polymeric structures.

In summary, polymeric cubosomes were prepared by a bottom-up method from self-assembly of PIL-BCPs. Under specific conditions within a narrow parameters range, the amphiphilic PAA-*b*-PILs self-assembled in THF/H_2_O mixtures to form nanoscale particles with cuboidal external morphology and internal bicontinuous cubic phase. This is comparable to the formation of crystals with different shape, where the lattice controls the formation of facets. To our knowledge, this has not been reported before for BCPs. In contrast to small molecules, the mesophasic structure is sensitive to small variations of the assembling conditions including polymer composition and concentration, temperature, the nature and content of the common solvent and precipitant (water). In particular, the mesophase structure is affected by the contribution of the surface energy, and as a consequence cuboid particles are mostly observed for the large objects. The temperature-dependent internal structure makes these cubosomes unique for application as thermosensitive polyelectrolytes and as model systems for drug delivery. The present study also revealed that the rational design of polymer structures and assembly conditions, as well as the presence of hierarchical interactions, is crucial to cubosome formation, providing guidelines for further investigations of special self-assembly phenomena and constructions of unique self-assembly structures.

## Methods

### Synthesis of P*t*BA-Br macroinitiator

CuBr_2_, tris(2-pyridylmethyl)amine, *tert*-butyl acrylate (*t*BA), ethyl 2-bromoisobutyrate, anisole and *N,N*-dimethylformamide were added to a Schlenk flask. The flask was then degassed by three freeze-pump-thaw cycles. While the contents were frozen in liquid nitrogen, the flask was back filled with nitrogen and CuBr was added. The flask was then degassed and back filled with nitrogen thrice. The flask was allowed to warm up to room temperature, and then placed in an oil bath at 90 °C. The desired amount of tin (II) 2-ethylhexanoate (Sn(EH)_2_) solution in anisole that has been purged with nitrogen was added to the flask, and an initial sample (*t*=0) was collected by syringe. At timed intervals, the reaction mixtures were taken for ^1^H NMR and GPC measurements. More desired amount of Sn(EH)_2_ solution in anisole was added in the flask during the polymerization if necessary. The polymerization was stopped by opening the flask and exposing the catalyst complex in the solution to air. The polymer was precipitated in cold methanol/water (4/1, v/v) mixture, and dried in vacuum at room temperature.

### Synthesis of P*t*BA-*b*-PIL diblock copolymers

The ionic monomer 1-(4-vinylbenzyl)-3-butylimidazolium bis(trifluoromethylsulfonyl)imide] (VBBI^+^Tf_2_N^−^) (ref. 54), CuBr_2_ in butyronitrile, *N*,*N*,*N*′,*N*′′,*N*′′-pentamethyldiethylenetriamine (PMDETA) and the P*t*BA-Br macroinitiator were added to a Schlenk flask. The flask was then degassed by three freeze-pump-thaw cycles. While the contents were frozen in liquid nitrogen, the flask was back filled with nitrogen and CuBr was added. The flask was then degassed and back filled with nitrogen thrice. The flask was allowed to warm up to room temperature and an initial sample (*t*=0) was collected by syringe. The flask was then placed in an oil bath at 90 °C. At timed intervals, the reaction mixtures were taken for ^1^H NMR and GPC measurements. The polymerization was stopped by opening the flask and exposing the catalyst complex in the solution to air. The polymer was precipitated in methanol/water (4/1, v/v) mixture, purified by dialysis (MWCO=3.5 kDa) against THF, and dried in vacuum at room temperature. The molar mass of the P*t*BA-*b*-PIL was obtained by comparing the integrated area of the NMR peaks of PIL block with that of P*t*BA block.

### Synthesis of PAA-*b*-PIL diblock copolymers

The *tert*-butyl groups in P*t*BA-*b*-PIL were removed via the treatment with TFA. Typically, P*t*BA-*b*-PIL (0.5 g) was dissolved in THF (2 ml), and then TFA (4 ml) in dichloromethane (2 ml) was added to the previous solution. The mixture was stirred for 1 day at room temperature. The solution was dried under vacuum, purified by dialysis and dried in vacuum at room temperature. Examination of the pKa values of TFA (*ca.* 0) and Tf_2_N^−^ (*ca.* −4 for the conjugate acid)^79^ indicates that the exchange between trifluoroacetate and Tf_2_N^−^ is unlikely (equilibrium constant *ca.* 10^−4^). Further prove comes from elemental analysis of PAA_45_-*b*-PIL_23:_ Elem. Anal. Calcd (%): C, 43.19; H, 4.40; F, 16.99; N, 6.26; S, 9.56; Found (%): C, 44.50; H, 4.39; F, 16.25; N, 6.04; S, 9.20.

### Self-assembling procedures

The desired amount of deionized water (1.6 ml, or other volumes for control samples) was added to a solution of PAA-*b*-PIL in THF (1.0 ml, 2 mg ml^−1^, or other concentrations for control samples) via a syringe needle under magnetic stirring. The water was added into the THF solution dropwise, quickly and continuously. The addition rate of water was 1.6 ml in 20 s (or other rates for control samples). The solution was allowed to stir for 1 h (or other desired time for chronological study) before TEM imaging.

### Data availability

The data that support the findings of this study are available from the corresponding author on request.

## 

## Additional information

**How to cite this article:** He, H. *et al*. Cubosomes from hierarchical self-assembly of poly(ionic liquid) block copolymers. *Nat. Commun.* **8,** 14057 doi: 10.1038/ncomms14057 (2017).

**Publisher's note:** Springer Nature remains neutral with regard to jurisdictional claims in published maps and institutional affiliations.

## Supplementary Material

Supplementary Information Supplementary Figures, Supplementary Tables, Supplementary Methods and Supplementary References

Supplementary Movie 1 The 4xspeed video showing the electron beam damage of the self-assembled aggregates of PAA_45_- b-PIL_23_ in TEM."
