# Supplementary material for: Cubosomes from hierarchical self-assembly of poly(ionic liquid) block copolymers
Source: Nat Commun. 2017 Jan 16;8:14057. doi: 10.1038/ncomms14057 (PMC5241804; doi:10.1038/ncomms14057)
Supplement: Supplementary Information — Supplementary Figures, Supplementary Tables, Supplementary Methods and Supplementary References [file ncomms14057-s1.pdf]

Supplementary Information

Supplementary Methods

Materials. 1-Butylimidazole (98%), bis(trifluoromethylsulfonyl)imide lithium salt ($LiTf_2N$, ≥99%) butyronitrile (99%), *tert*-Butyl acrylate (*t*BA, 98%), ethyl 2-bromoisobutyrate (EBiB, 98%), *N*,*N*,*N*′,*N*′′,*N*′′-pentamethyldiethylenetriamine (PMDETA, 99%), tin(II) 2-ethylhexanoate ($Sn(EH)_2$, 95%), and trifluoroacetic acid (TFA, 99%) were purchased from Aldrich. 4-Vinylbenzyl chloride (90%) and styrene (≥99%) were purchased from Aldrich and purified by passing over a column of basic alumina to remove the inhibitor. CuBr and $CuBr_2$ were purchased from Aldrich in the highest available purity. Tris(2-pyridylmethyl)amine (TPMA) was purchased from ATRP Solutions or synthesized according to previously published procedures.[1] All solvents and chemicals are of reagent quality and were used as received unless special explanation. The ionic monomer, 1-(4-vinylbenzyl)-3-butylimidazolium bis(trifluoromethylsulfonyl)imide] ($VBBI^+Tf_2N^-$), was synthesized according to previously published procedures.[2]

Instrumentation.

^{1}H nuclear magnetic resonance (NMR) measurements were performed on a Bruker Avance 300 or 500 MHz spectrometer.

Molecular weight and molecular weight distribution (M_w/M_n) values were determined by gel permeation chromatography (GPC). The GPC system used a Waters 515 HPLC pump and a Waters 2414 refractive index detector using PSS columns (Styrogel 10^2, 10^3, 10^5 Å) with tetrahydrofuran (THF) or solute-containing THF as the eluent at a flow rate of 1 mL/min at 35 ℃. The sample was diluted with THF and filtered through a column of neutral alumina to remove the catalyst, and then filtered through 0.2 μm polytetrafluoroethylene (PTFE) membrane filter before injecting into GPC columns (inject volume: 40 μL) with diphenyl ether (DPE) as the flow marker.

Elemental analyses were performed on an Exeter Analytical CE440 elemental analyser.

Transmission electron microscopy (TEM) analysis was conducted using a Hitachi H-7100 transmission electron microscope (Hitachi High Technologies America) operating at 75 kV. A small drop of solution containing the sample was placed on a carbon coated copper grid. After several seconds, the drop was removed by blotting with filter paper. The sample that remained on the grid was allowed to

dry before inserting the grid into the microscope. Digital images were obtained using AMT Advantage 10 CCD Camera System and NIH Image software.

Cryogenic transmission electron microscopy (cryo-TEM) micrographs were taken on a Carl Zeiss Libra 120 Microscope (Oberkochen, Germany). Cryogenic TEM samples were prepared by plunge freezing of aqueous dispersion using plasma-treated lacey grids and a vitrobot system. The vitrified specimens were transferred to a cryo-holder and observed at 120 kV acceleration voltage at about -170 ^{o}C.

Scanning electron microscopy (SEM) analysis was conducted using a Philips XL30 field emission scanning electron microscope. Au particles were sputtered on the surface of the sample on silicon wafers with a sputter coater (Pelco SC-6) before SEM imaging. For EDX analysis, cubosomes were deposited on a Si-wafer and after drying analyzed by ultra-high resolution SEM (UHR FE-SEM SU9000, lateral resolution 0.4 nm at 30 kV, 1.2 nm at 1 kV) equipped with an EDX spectrometer (Oxford Instruments X-max 80M detectors).

Particle size and zeta potential were measured using a Zetasizer Nano from Malvern Instruments.

Low angle X-ray diffraction measurement was performed in-house using an Empyrean setup from PANalytical using CuK_α radiation. A parabolic graded multilayer system converted the divergent line source (12×0.04 mm) into an almost parallel beam with divergence <0.03°. After transmission from the capillary the intensity was collected by a pixel detector (256×256 pixels of 55 μm, all connected as a 0-D detector) as a function of the scattering vector q_z=(4π/λ)sinθ, 2θ being the scattering angle. The latter was fixed at twice the incident angle θ, which sets q_z along the film normal. The SAXS experiments were performed at beamline ID02 of ESRF.

Supplementary Figures

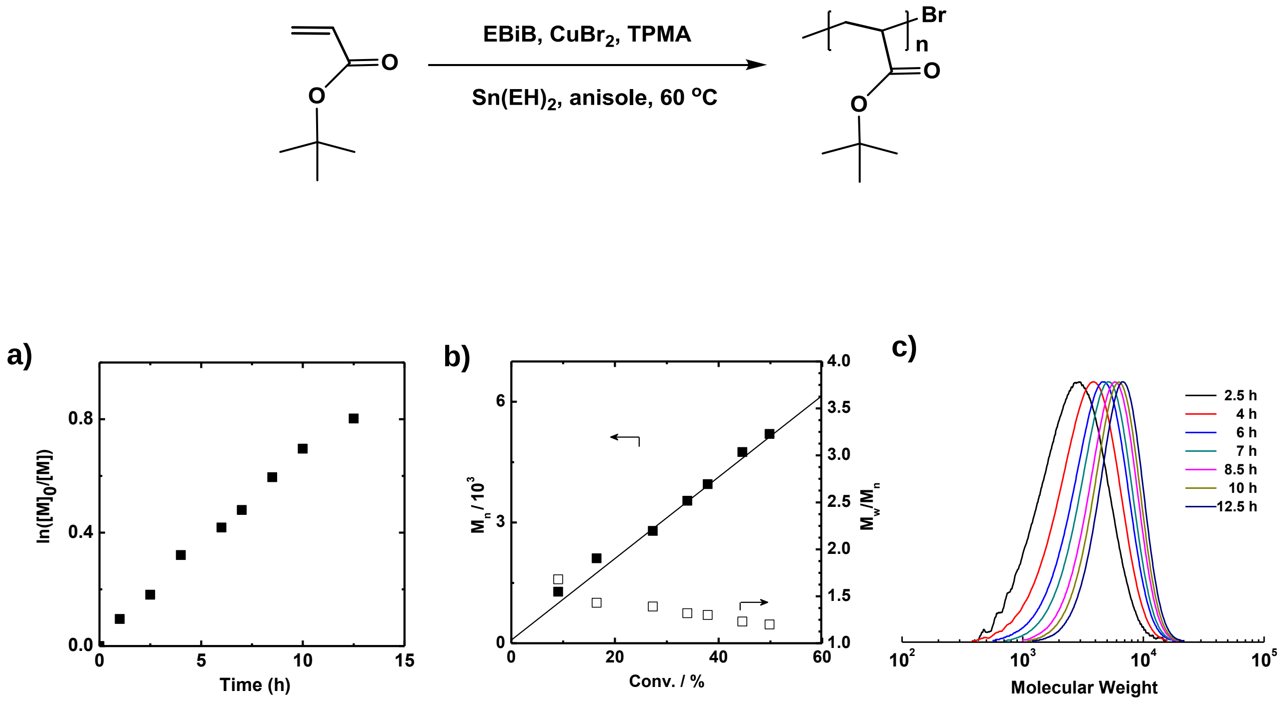

Supplementary Figure 1. Synthesis of P*t*BA macroinitiator (P*t*BA$_{45}$-Br) by ARGET ATRP. Kinetic plot of ln([M]$_0$/[M]) *vs* time (a), plot of M_n and M_w/M_n *vs* conversion (b), and GPC traces (c) for ARGET ATRP of *tert*-butyl acrylate with 150 ppm Cu. Conditions: [*t*BA]$_0$/[EBiB]$_0$/[$CuBr_2$]$_0$/[TPMA]$_0$/[Sn(EH)$_2$]$_0$ = 75/1/0.01125/0.03/0.03, anisole/DMF/*t*BA = 1/0.3/3 (v/v/v), 60 °C. Sn(EH)$_2$ was added in separated portions during the polymerization: 1/3 at 0 h, 1/3 at 1.5 h, and 1/3 at 6.5 h. At 12.5 h, *conv.* = 55.2%. P*t*BA-Br: DP = 45, M_n = 5950, PDI = 1.17.

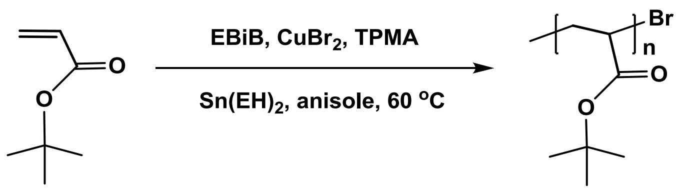

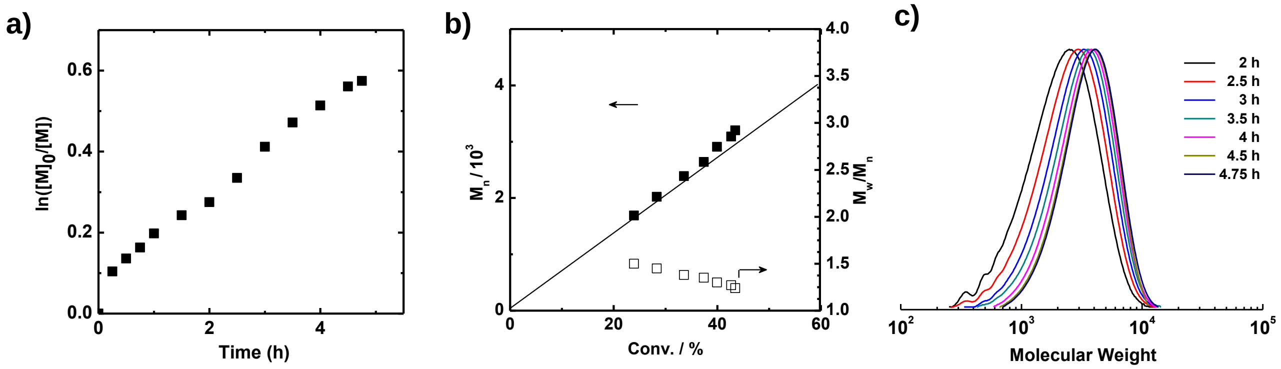

Supplementary Figure 2. Synthesis of P*t*BA macroinitiator (P*t*BA$_{23}$-Br) by ARGET ATRP. Kinetic plot of ln([M]$_0$/[M]) *vs* time (a), plot of M_n and M_w/M_n *vs* conversion (b), and GPC traces (c) for ARGET ATRP of *tert*-butyl acrylate with 150 ppm Cu. Conditions: [*t*BA]$_0$/[EBiB]$_0$/[CuBr$_2$]$_0$/[TPMA]$_0$/[Sn(EH)$_2$]$_0$ = 50/1/0.0075/0.05/0.03, anisole/DMF/*t*BA = 1/0.3/3 (v/v/v), 60 °C. Sn(EH)$_2$ was added in separated portions during the polymerization: 1/3 at 0 h, 1/3 at 2 h, and 1/3 at 3 h. At 4.75 h, *conv.* = 43.7%. P*t*BA-Br: DP = 23, M_n = 3200, PDI = 1.22.

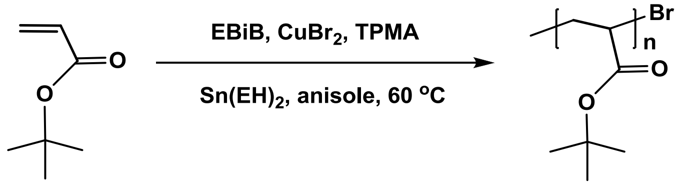

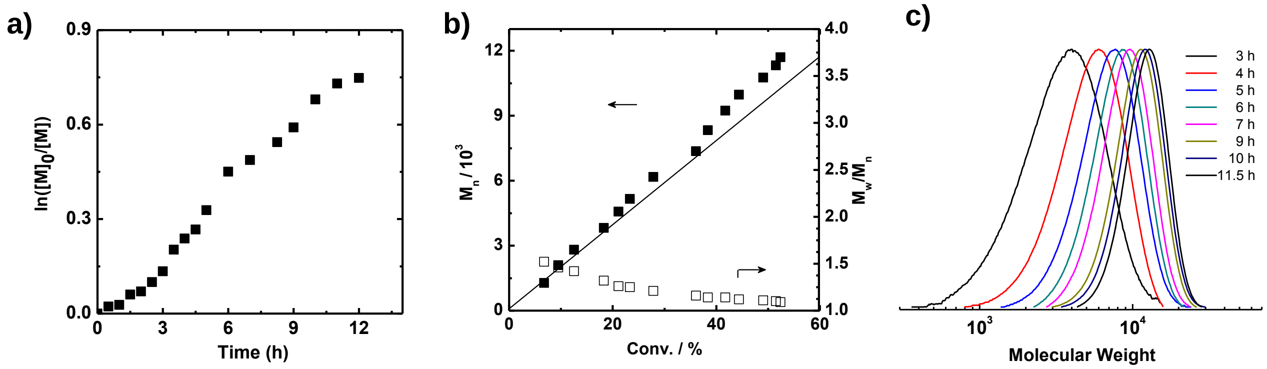

Supplementary Figure 3. Synthesis of P*t*BA macroinitiator (P*t*BA$_{90}$-Br) by ARGET ATRP. Kinetic plot of ln([M]$_0$/[M]) *vs* time (a), plot of M_n and M_w/M_n *vs* conversion (b), and GPC traces (c) for ARGET ATRP of *tert*-butyl acrylate with 150 ppm Cu. Conditions: [*t*BA]$_0$/[EBiB]$_0$/[CuBr$_2$]$_0$/[TPMA]$_0$/[Sn(EH)$_2$]$_0$ = 150/1/0.0225/0.05/0.07, anisole/DMF/*t*BA = 1/0.3/3 (v/v/v), 60 °C. Sn(EH)$_2$ was added in separated portions during the polymerization: 2/7 at 0 h, 1/7 at 1 h, 1/7 at 2 h, 1/7 at 3 h, 1/7 at 4.5 h, and 1/7 at 7.75 h. At 11.5 h, *conv.* = 52.7%. P*t*BA-Br: DP = 90, M_n = 11780, PDI = 1.09.

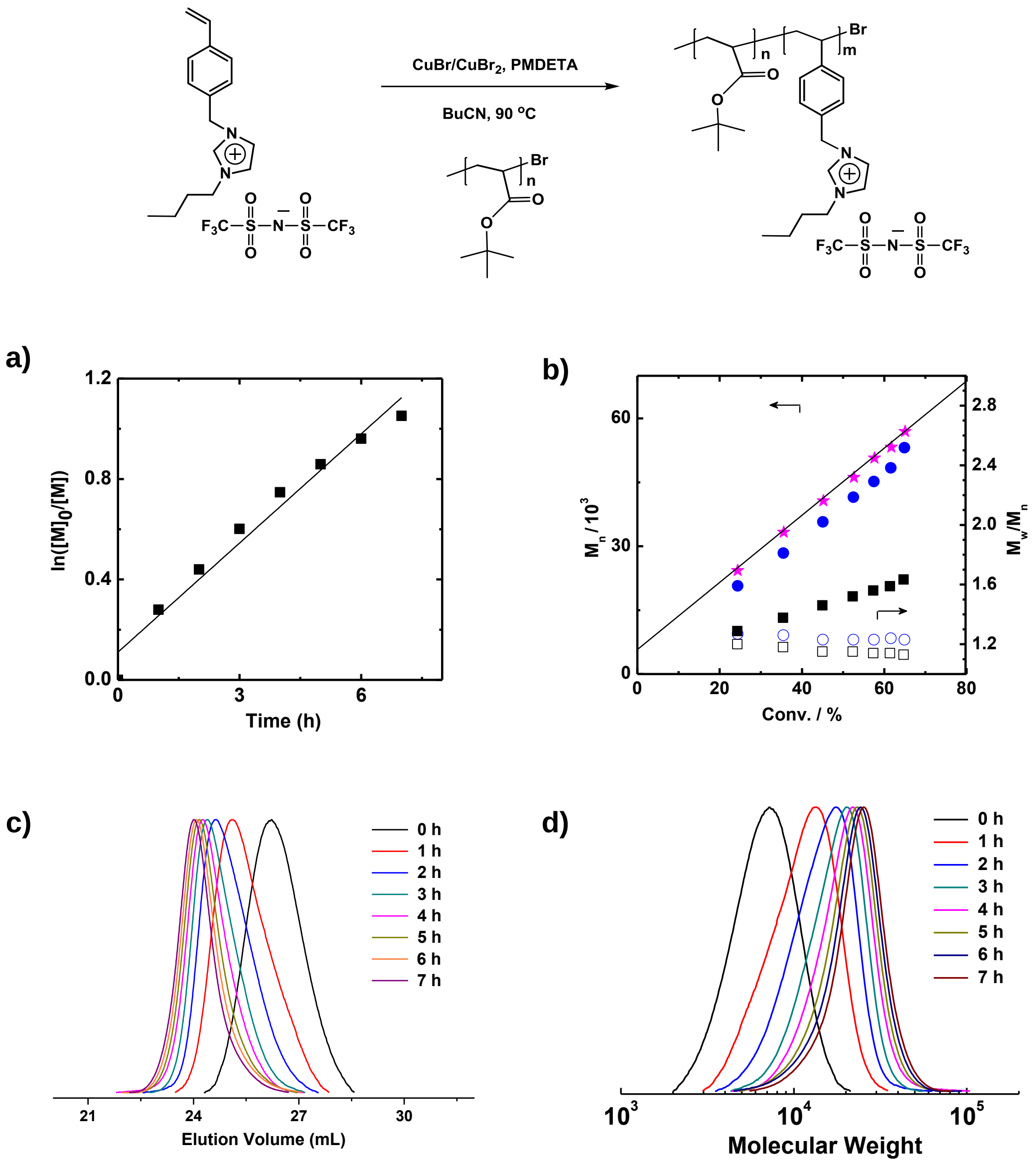

Supplementary Figure 4. Synthesis of P*t*BA$_{45}$-*b*-PIL$_{98}$ by ATRP using P*t*BA$_{45}$-Br macroinitiator. Polymerization results for Entry 3 in Supplementary Table 1. Kinetic plot of ln([M]$_0$/[M]) *vs* time (a), plot of M_n and M_w/M_n *vs* conversion calibrated using PS (black squares) and polyVBBI$^+$Tf$_2$N$^-$$_{RAFT}$ standards (blue circles), and M_n obtained by NMR (pink stars) (b), and GPC traces (in THF containing 10 mM LiTf$_2$N and 10 mM 1-butylimidazole) (c,d) for ATRP of VBBI$^+$Tf$_2$N$^-$. Conditions: [VBBI$^+$Tf$_2$N$^-$]$_0$/[P*t*BA$_{45}$-Br]$_0$/[CuBr]$_0$/[CuBr$_2$]$_0$/[PMDETA]$_0$ = 150/1/2.85/0.15/3, VBBI$^+$Tf$_2$N$^-$/BuCN = 1/1 (w/w), 90 °C. At 7 h, *conv.* = 65.1%.

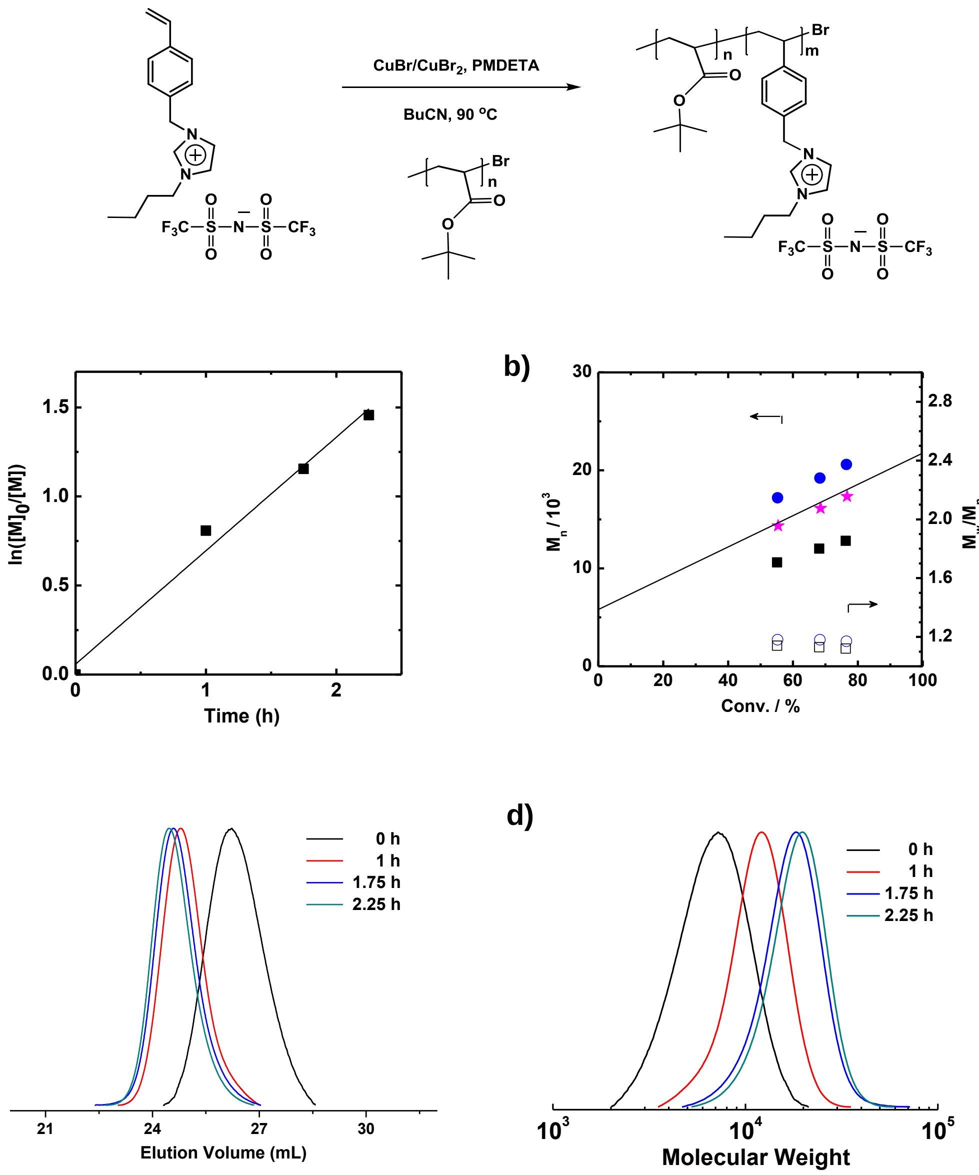

Supplementary Figure 5. Synthesis of P*t*BA$_{45}$-*b*-PIL$_{23}$ by ATRP using P*t*BA$_{45}$-Br macroinitiator. Polymerization results for Entry 1 in Supplementary Table 1. Kinetic plot of ln([M]$_0$/[M]) *vs* time (a), plot of M_n and M_w/M_n *vs* conversion calibrated using PS (black squares) and polyVBBI$^+$Tf$_2$N$^-_{RAFT}$ standards (blue circles), and M_n obtained by NMR (pink stars) (b), and GPC traces (in THF containing 10 mM LiTf$_2$N and 10 mM 1-butylimidazole) calibrated using PS (c,d) for ATRP of VBBI$^+$Tf$_2$N$^-$. Conditions: [VBBI$^+$Tf$_2$N$^-$]$_0$/[P*t*BA$_{45}$-Br]$_0$/[CuBr]$_0$/[CuBr$_2$]$_0$/[PMDETA]$_0$ = 30/1/0.95/0.05/1, VBBI$^+$Tf$_2$N$^-$/BuCN = 1/1 (w/w), 90 °C. At 2.25 h, *conv.* = 76.7%.

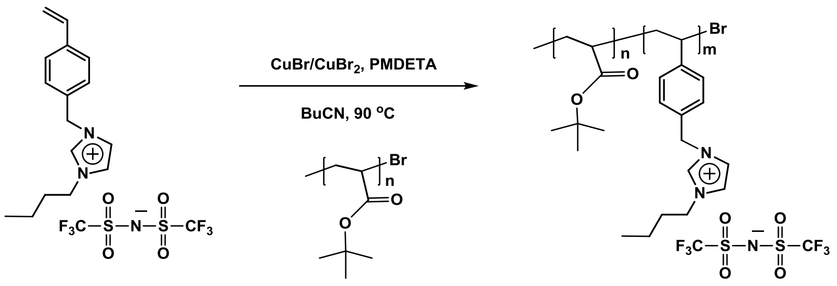

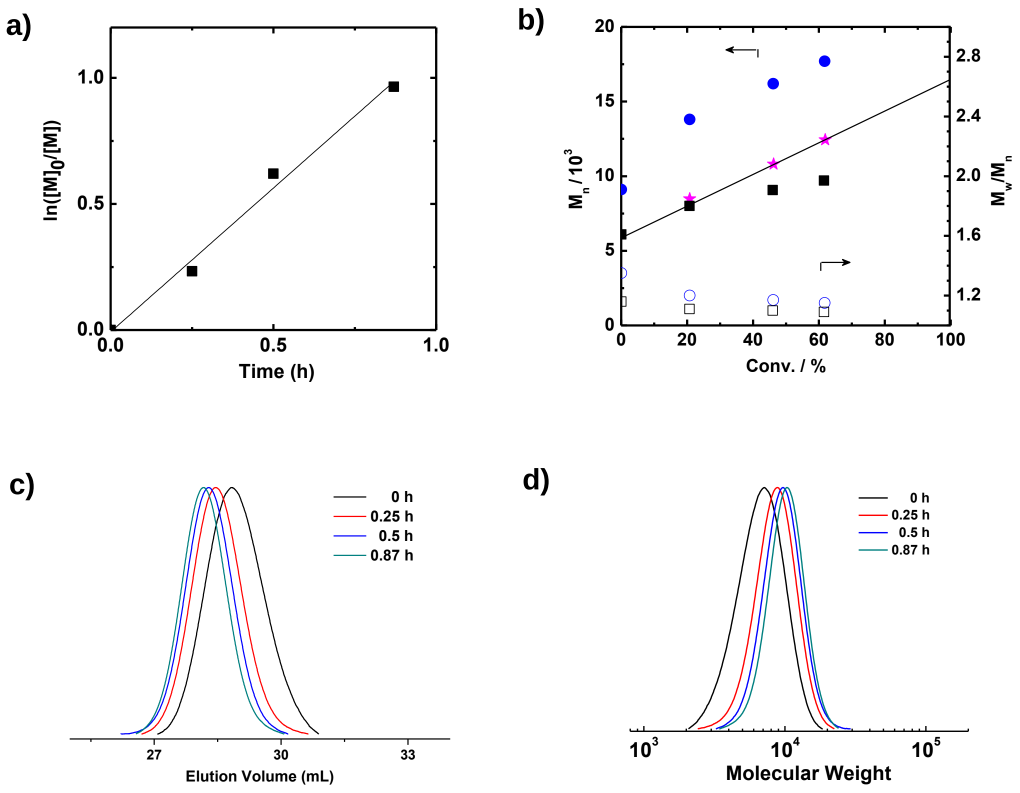

Supplementary Figure 6. Synthesis of P*t*BA$_{45}$-*b*-PIL$_{12}$ by ATRP using P*t*BA$_{45}$-Br macroinitiator. Polymerization results for Entry 2 in Supplementary Table 1. Kinetic plot of ln([M]$_0$/[M]) *vs* time (a), plot of M_n and M_w/M_n *vs* conversion calibrated using PS (black squares) and polyVBBI$^+$Tf$_2$N$^-_{RAFT}$ standards (blue circles), and M_n obtained by NMR (pink stars) (b), and GPC traces (in THF containing 10 mM LiTf$_2$N and 10 mM 1-butylimidazole) calibrated using PS (c,d) for ATRP of VBBI$^+$Tf$_2$N$^-$. Conditions: [VBBI$^+$Tf$_2$N$^-$]$_0$/[P*t*BA$_{45}$-Br]$_0$/[CuBr]$_0$/[CuBr$_2$]$_0$/[PMDETA]$_0$ = 20/1/0.95/0.05/1, VBBI$^+$Tf$_2$N$^-$/BuCN = 1/1 (w/w), 90 °C. At 0.87 h, *conv.* = 61.9%.

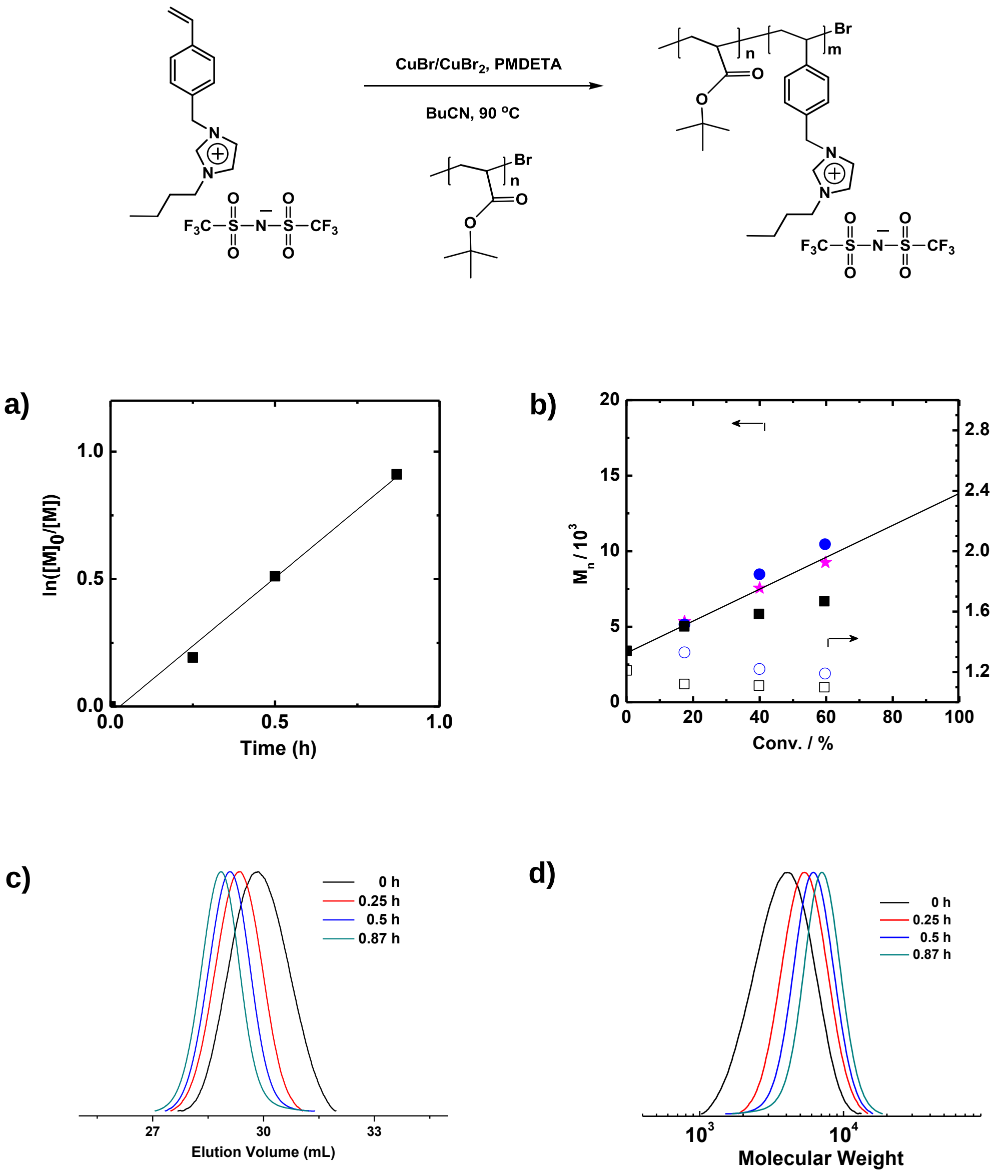

Supplementary Figure 7. Synthesis of P*t*BA$_{23}$-*b*-PIL$_{12}$ by ATRP using P*t*BA$_{23}$-Br macroinitiator. Polymerization results for Entry 4 in Supplementary Table 1. Kinetic plot of ln([M]$_0$/[M]) *vs* time (a), plot of M_n and M_w/M_n *vs* conversion calibrated using PS (black squares) and polyVBBI$^+$Tf$_2$N$^-$$_{RAFT}$ standards (blue circles), and M_n obtained by NMR (pink stars) (b), and GPC traces (in THF containing 10 mM LiTf$_2$N and 10 mM 1-butylimidazole) calibrated using PS (c,d) for ATRP of VBBI$^+$Tf$_2$N$^-$. Conditions: [VBBI$^+$Tf$_2$N$^-$]$_0$/[P*t*BA$_{23}$-Br]$_0$/[CuBr]$_0$/[CuBr$_2$]$_0$/[PMDETA]$_0$ = 20/1/0.95/0.05/1, VBBI$^+$Tf$_2$N$^-$/BuCN = 1/1 (w/w), 90 °C. At 0.87 h, *conv.* = 59.8%.

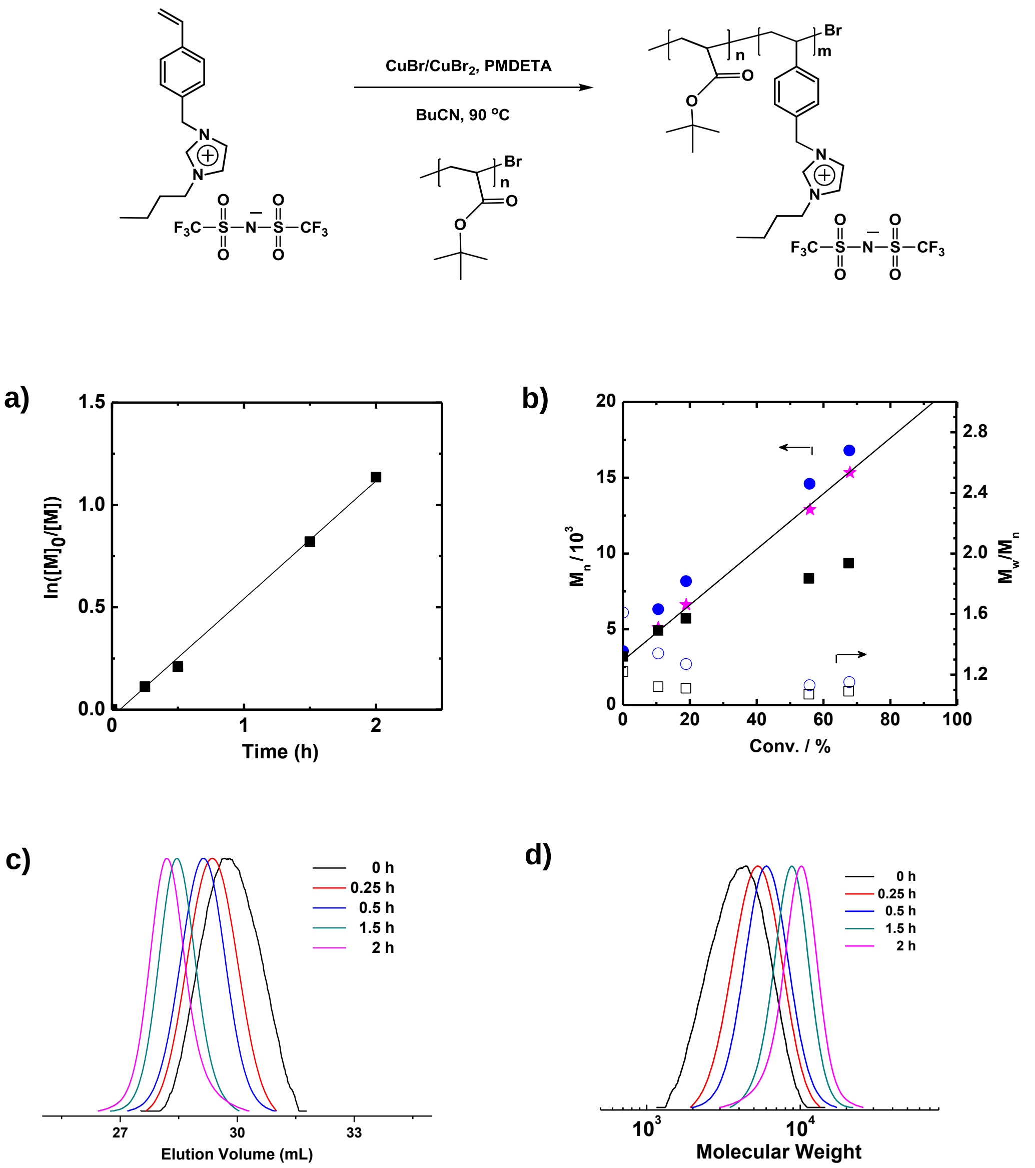

Supplementary Figure 8. Synthesis of P*t*BA$_{23}$-*b*-PIL$_{23}$ by ATRP using P*t*BA$_{23}$-Br macroinitiator. Polymerization results for Entry 5 in Supplementary Table 1. Kinetic plot of ln([M]$_0$/[M]) *vs* time (a), plot of M_n and M_w/M_n *vs* conversion calibrated using PS (black squares) and polyVBBI$^+$Tf$_2$N$^-_{RAFT}$ standards (blue circles), and M_n obtained by NMR (pink stars) (b), and GPC traces (in THF containing 10 mM LiTf$_2$N and 10 mM 1-butylimidazole) calibrated using PS (c,d) for ATRP of VBBI$^+$Tf$_2$N$^-$. Conditions: [VBBI$^+$Tf$_2$N$^-$]$_0$/[P*t*BA$_{23}$-Br]$_0$/[CuBr]$_0$/[CuBr$_2$]$_0$/[PMDETA]$_0$ = 35/1/0.95/0.05/1, VBBI$^+$Tf$_2$N$^-$/BuCN = 1/1 (w/w), 90 °C. At 2 h, *conv.* = 67.9%.

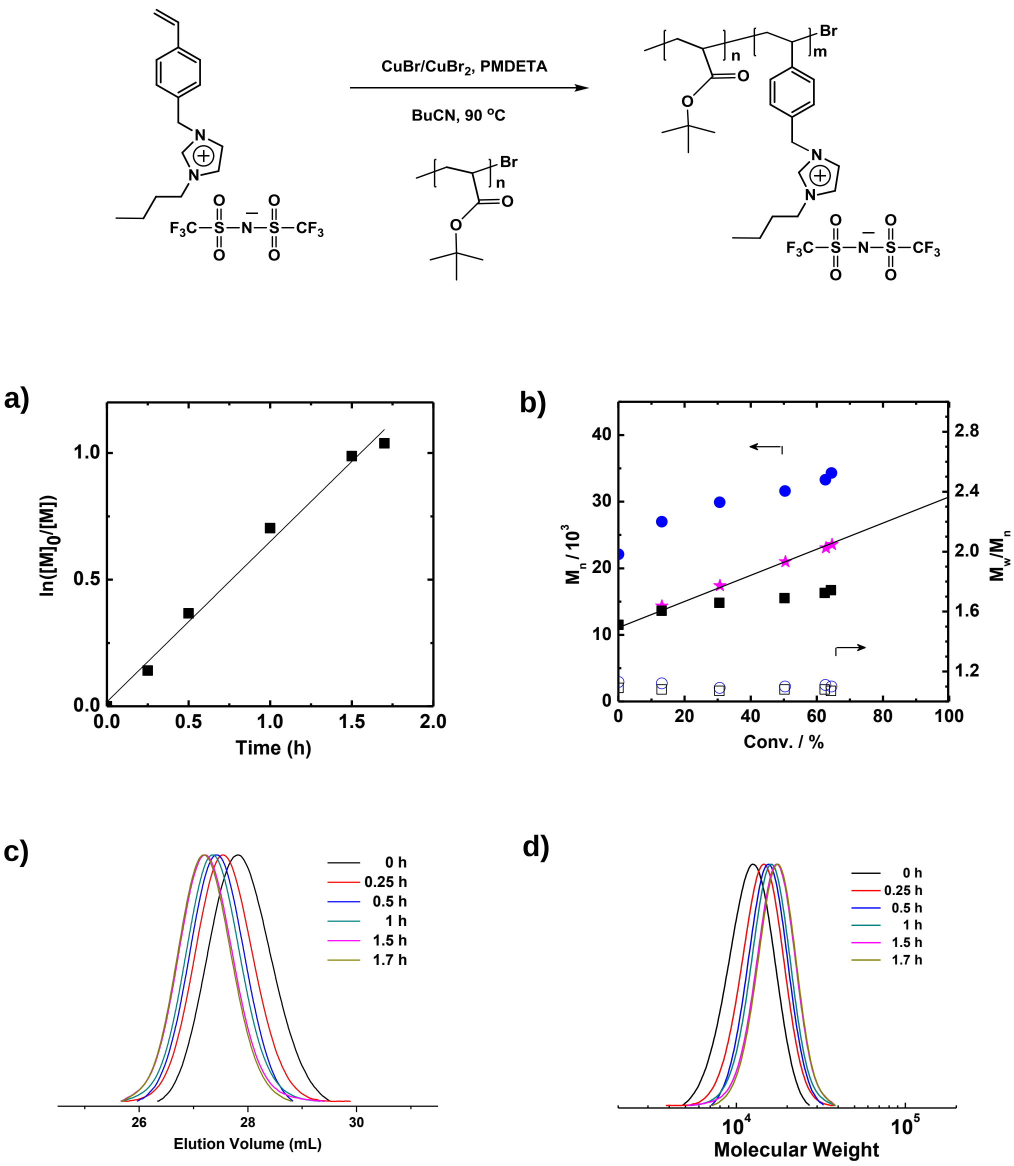

Supplementary Figure 9. Synthesis of P*t*BA$_{90}$-*b*-PIL$_{23}$ by ATRP using P*t*BA$_{90}$-Br macroinitiator. Polymerization results for Entry 6 in Supplementary Table 1. Kinetic plot of ln([M]$_0$/[M]) *vs* time (a), plot of M_n and M_w/M_n *vs* conversion calibrated using PS (black squares) and polyVBBI$^+$Tf$_2$N$^-_{RAFT}$ standards (blue circles), and M_n obtained by NMR (pink stars) (b), and GPC traces (in THF containing 10 mM LiTf$_2$N and 10 mM 1-butylimidazole) calibrated using PS (c,d) for ATRP of VBBI$^+$Tf$_2$N$^-$. Conditions: [VBBI$^+$Tf$_2$N$^-$]$_0$/[P*t*BA$_{90}$-Br]$_0$/[CuBr]$_0$/[CuBr$_2$]$_0$/[PMDETA]$_0$ = 20/1/0.95/0.05/1, VBBI$^+$Tf$_2$N$^-$/BuCN = 1/1 (w/w), 90 °C. At 1.7 h, *conv.* = 64.6%.

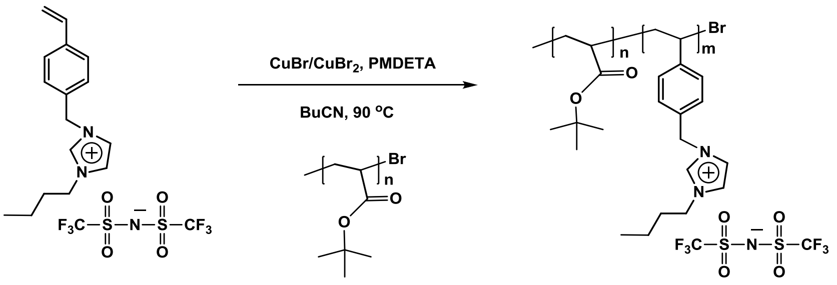

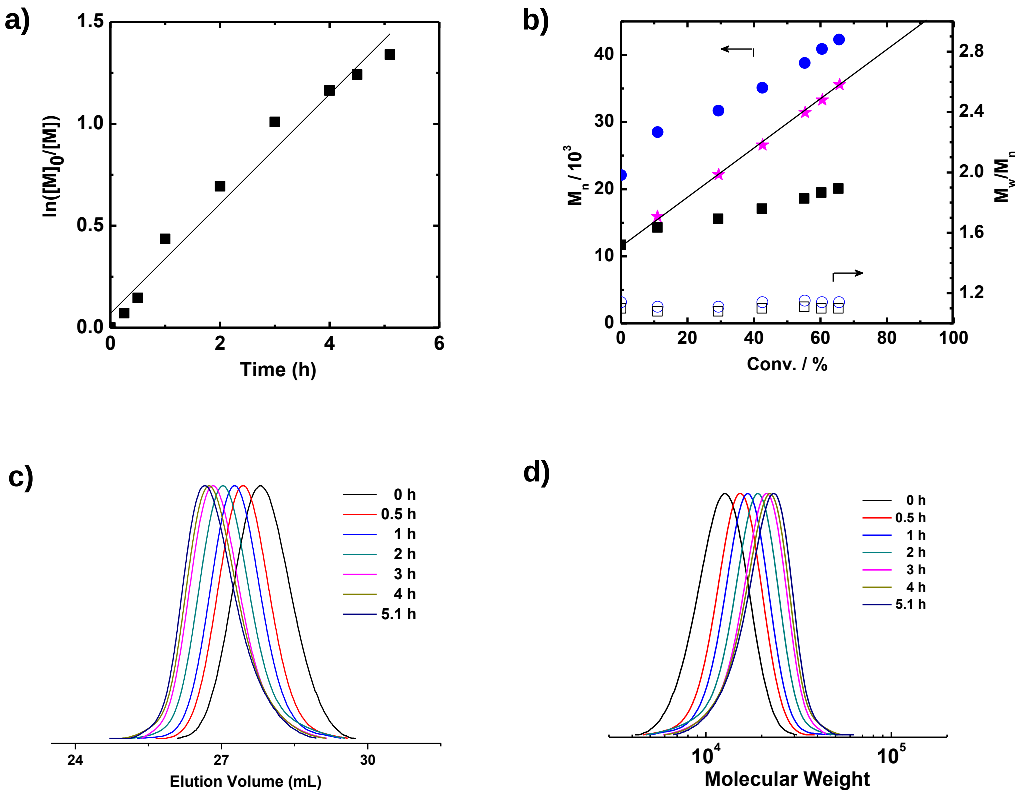

Supplementary Figure 10. Synthesis of P*t*BA$_{90}$-*b*-PIL$_{46}$ by ATRP using P*t*BA$_{90}$-Br macroinitiator. Polymerization results for Entry 7 in Supplementary Table 1. Kinetic plot of ln([M]$_0$/[M]) *vs* time (a), plot of M_n and M_w/M_n *vs* conversion calibrated using PS (black squares) and polyVBBI$^+$Tf$_2$N$^-_{RAFT}$ standards (blue circles), and M_n obtained by NMR (pink stars) (b), and GPC traces (in THF containing 10 mM LiTf$_2$N and 10 mM 1-butylimidazole) calibrated using PS (c,d) for ATRP of VBBI$^+$Tf$_2$N$^-$. Conditions: [VBBI$^+$Tf$_2$N$^-$]$_0$/[P*t*BA$_{90}$-Br]$_0$/[CuBr]$_0$/[CuBr$_2$]$_0$/[PMDETA]$_0$ = 70/1/0.95/0.05/1, VBBI$^+$Tf$_2$N$^-$/BuCN = 1/1 (w/w), 90 °C. At 5.1 h, *conv.* = 65.8%.

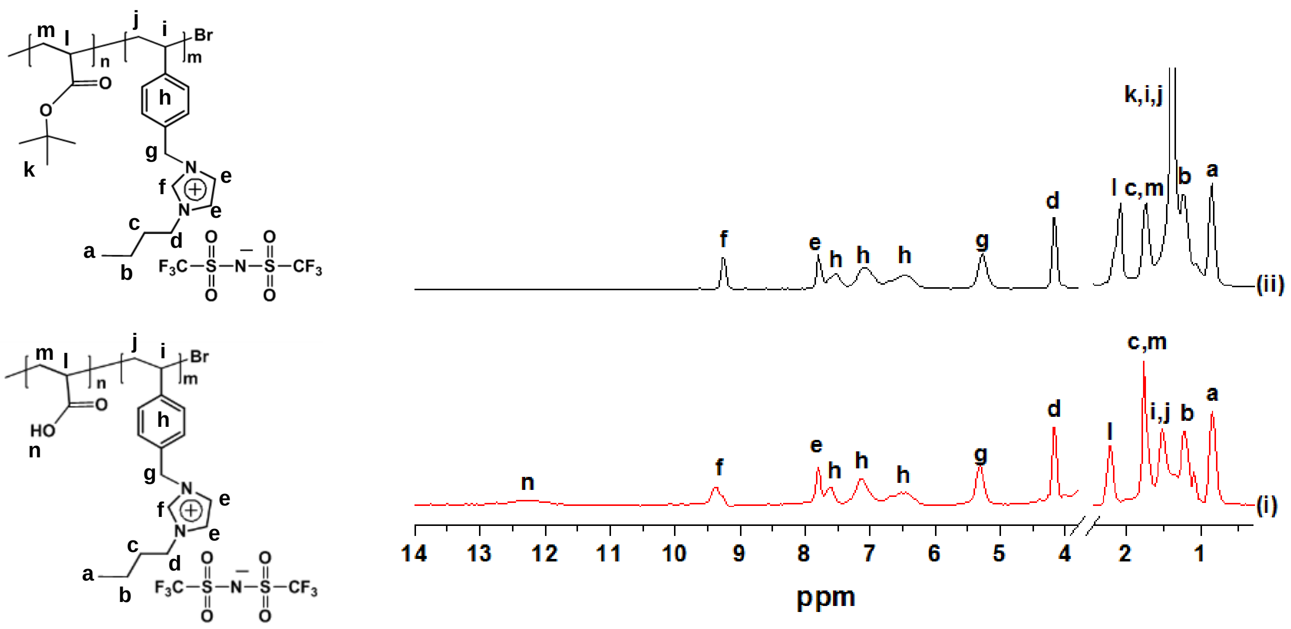

Supplementary Figure 11. ^{1}HNMR spectra of PAA_{45}-*b*-PIL_{23} (i) and P*t*BA_{45}-*b*-PIL_{23} (ii).

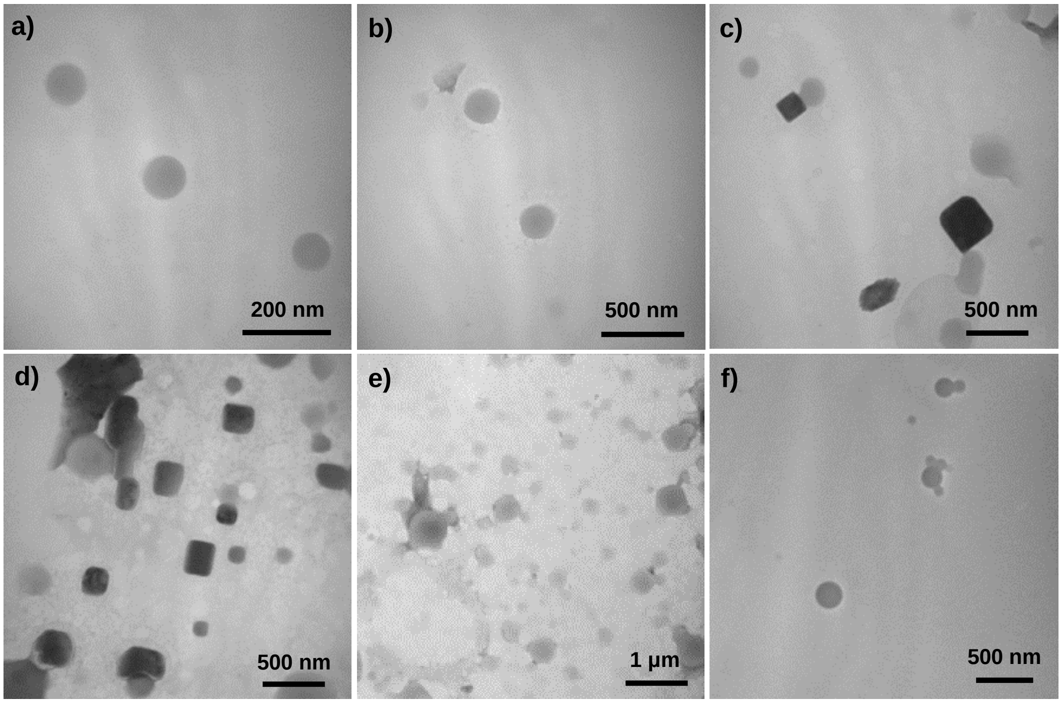

Supplementary Figure 12. TEM images of the self-assembled aggregates of PAA_{45}-*b*-PIL_{23}. Self-assembling conditions: 2 mg/mL THF solution of PAA_{45}-*b*-PIL_{23}, water/THF volume ratios R = 1.2 (a), 1.4 (b), 1.5 (c), 1.6 (d), 1.7 (e), and 1.8 (f).

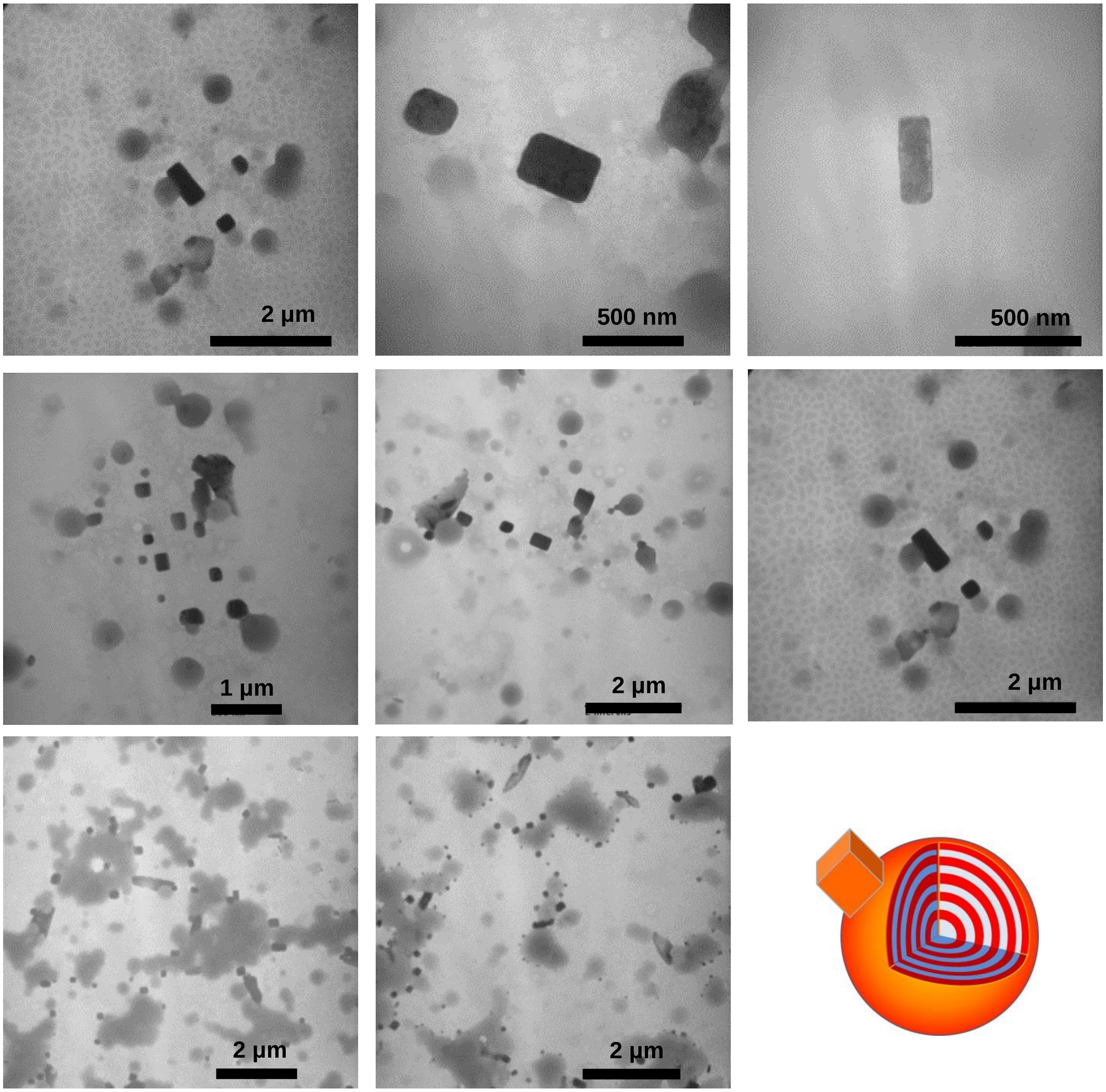

Supplementary Figure 13. TEM images of the self-assembled aggregates of PAA_{45}-*b*-PIL_{23} (2 mg/mL THF solution) with the adding of water at water/THF volume ratios (R) = 1.6. The lower right image shows the schematic illustration of growing cubosomes after formation of multilamellar vesicles (MLVs).

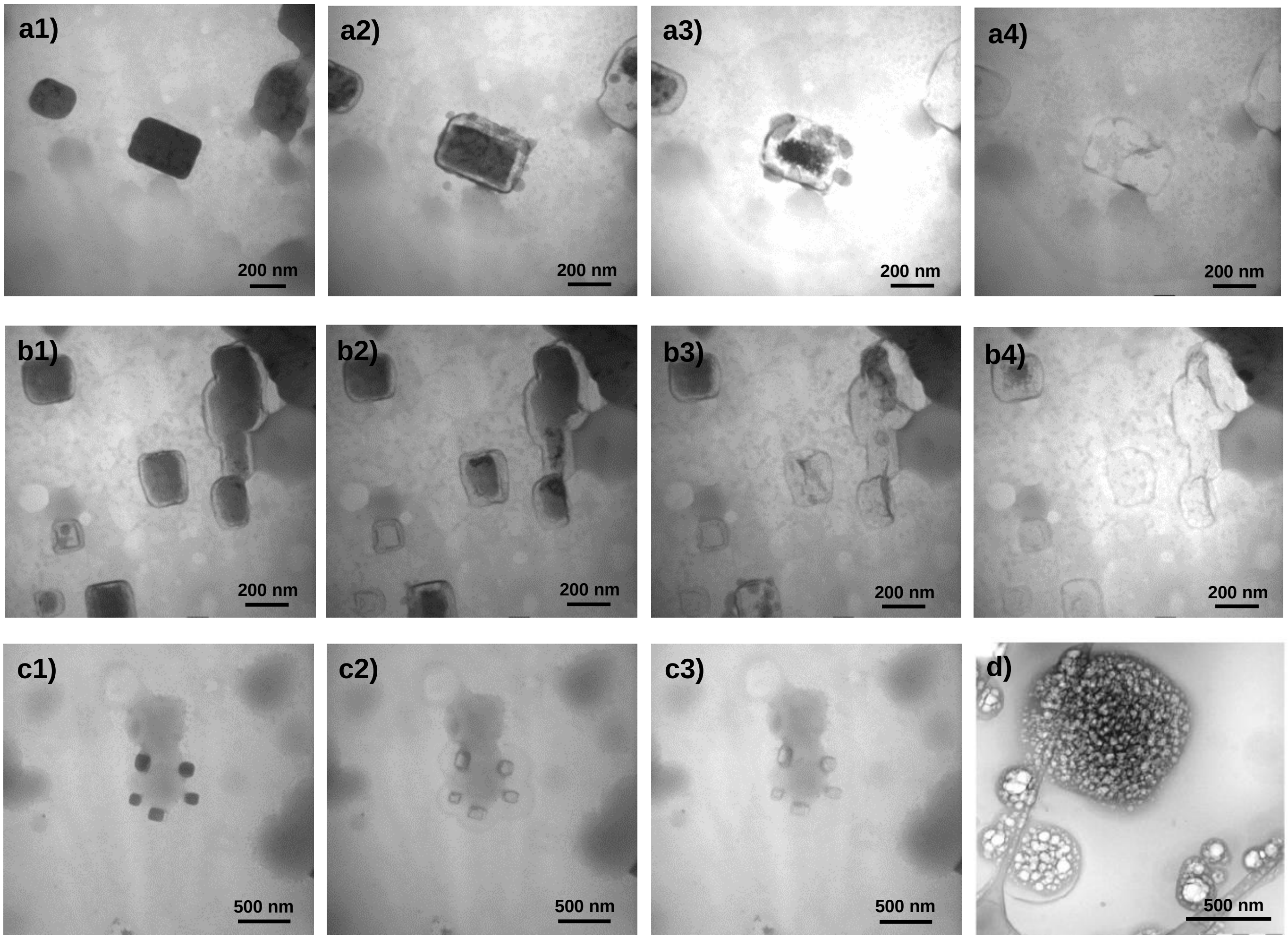

Supplementary Figure 14. TEM images (a-c) and cryo-TEM image (d) showing the electron beam damage (melting and degradation) of the self-assembled aggregates of PAA_{45}-*b*-PIL_{23} (2 mg/mL THF solution) with the adding of water at water/THF volume ratios (*R*) = 1.6. The images a1-a4, b1-b4, and c1-c3 were taken at the same area of the samples at elongated time of exposure, respectively.

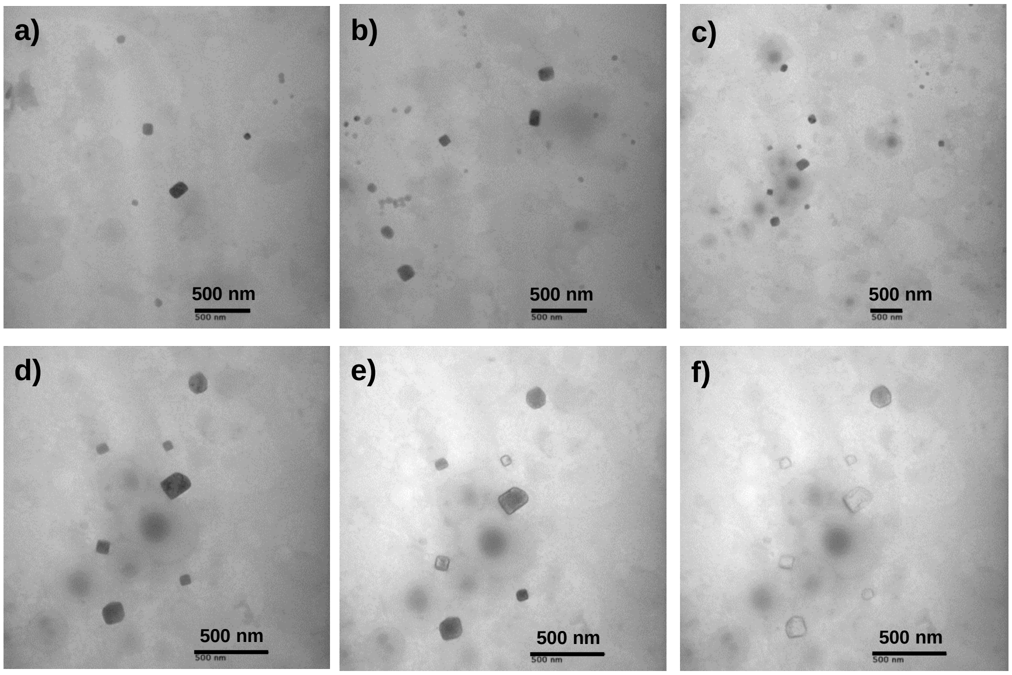

Supplementary Figure 15. TEM images of the self-assembled aggregates of PAA_{45}-*b*-PIL_{23} (2 mg/mL THF solution) with the adding of water with water/THF volume ratio (*R)* = 1.6. $PtBA_{45}$-*b*-PIL_{23} was synthesized by ARGET ATRP with 100 ppm Cu. Conditions: $[VBBI^{+}Tf_2N^{-}]_0/[PtBA_{45}\text{-}Br]_0/[CuBr_2]_0/[TPMA]_0/[Sn(EH)_2]_0$ = 60/1/0.006/0.4/0.03, $BuCN/VBBI^{+}Tf_2N^{-}$ = 1/1 (w/w), 90 ℃. Images a-c were taken at different areas of the sample. Images d-f were taken at the same area of the sample at elongated time of exposure, showing the electron beam damage of the self-assembled aggregates.

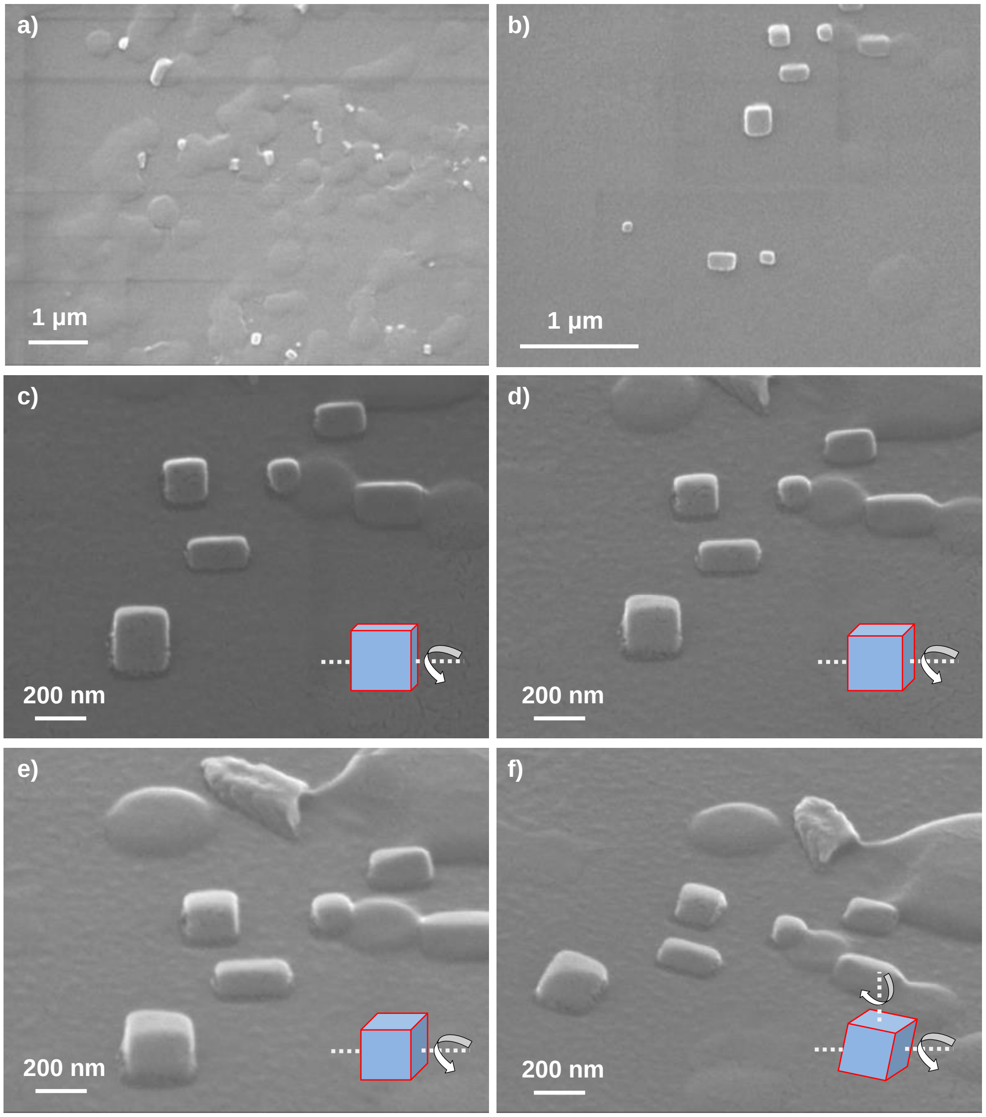

Supplementary Figure 16. Tilted SEM images of the self-assembled aggregates of PAA_{45}-*b*-PIL_{23} on silicon wafers at (a,b) 0°, (c) +30°, (d) +45°, and (e,f) +55° tilting angle. Additional rotation angle of +30° was applied in (f). Au particles were sputtered on the surface of the sample before SEM imaging. Self-assembling conditions: 2 mg/mL THF solution of PAA_{45}-*b*-PIL_{23}, water/THF volume ratio (*R)* = 1.6. The edges of the cuboidal particles gradually became brighter with increasing of the tilting angles from 0° to 55°, which was due to the edge charging effect. When an additional rotation angle of 30° was applied, the side surfaces of the cuboidal particles were also visualized in the SEM image.

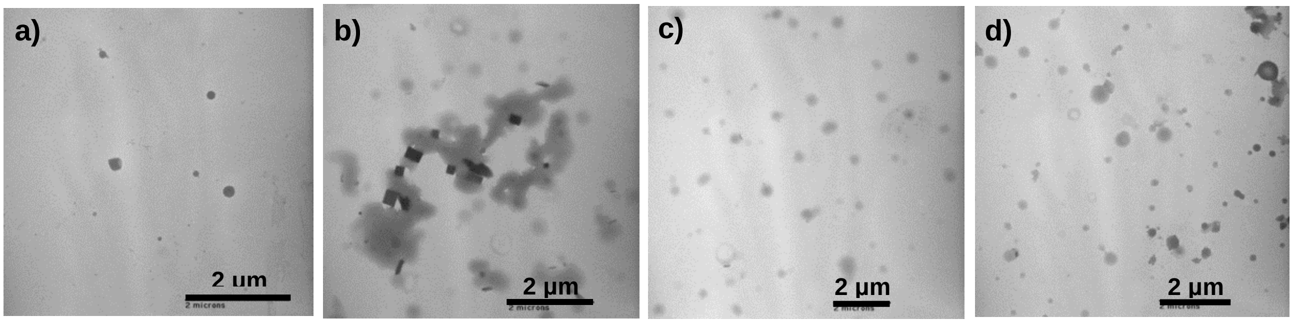

Supplementary Figure 17. TEM images of the self-assembled aggregates of PAA_{45}-*b*-PIL_{23} (2 mg/mL THF solution) with water/THF volume ratio R = 1.6 and the additon of a) aqueous NaOH solution (pH = 9), b) aqueous HCl solution (pH = 5), and c) aqueous HCl solution (pH = 1), and d) aqueous NaCl solution (0.1 M).

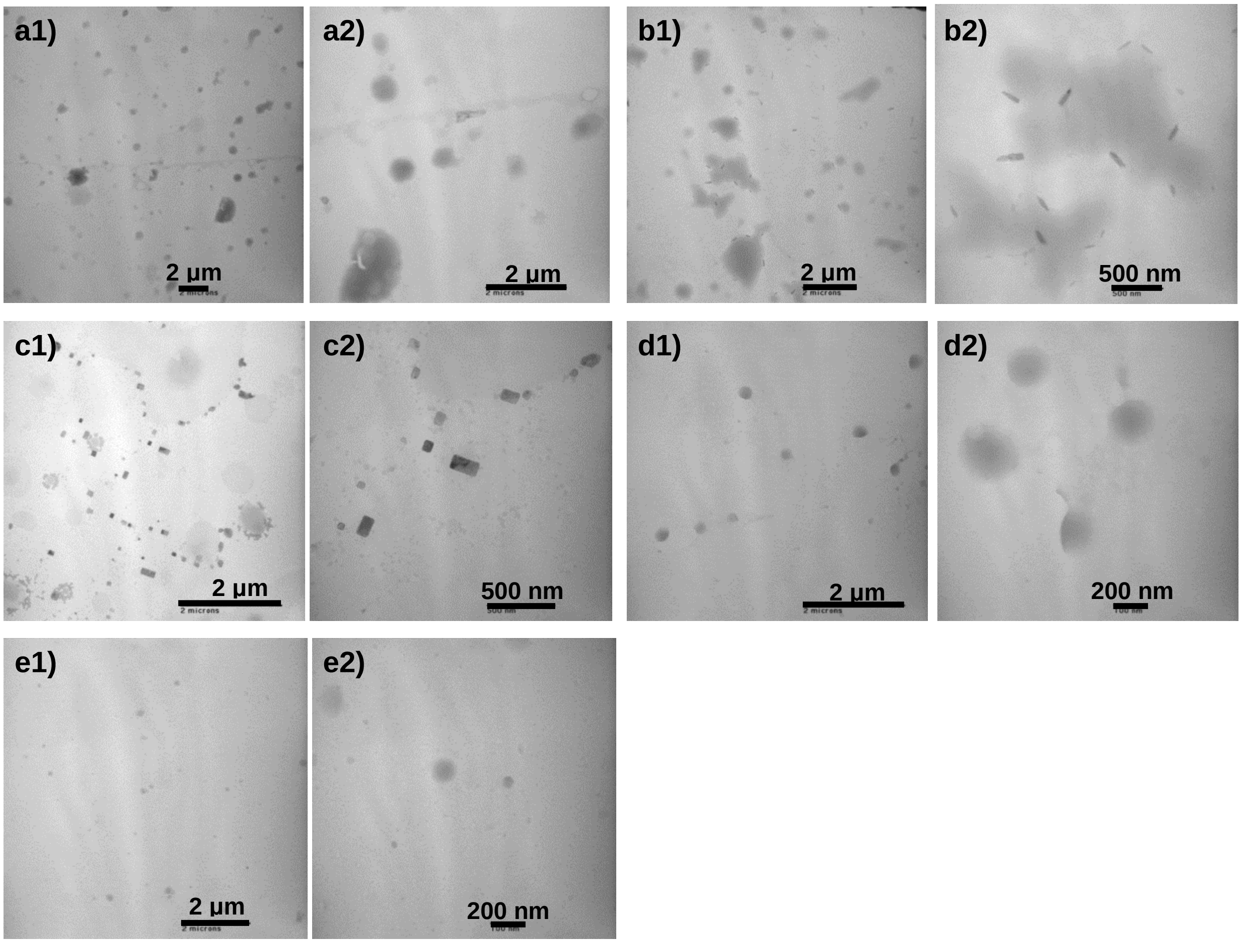

Supplementary Figure 18. TEM images of the self-assembled aggregates of PAA_{45}-*b*-PIL_{23} at water/THF volume ratio (R) = 1.6 with different polymer concentrations: a) 20 mg/mL, b) 10 mg/mL, c) 5 mg/mL, d) 1 mg/mL, and e) 0.5 mg/mL.

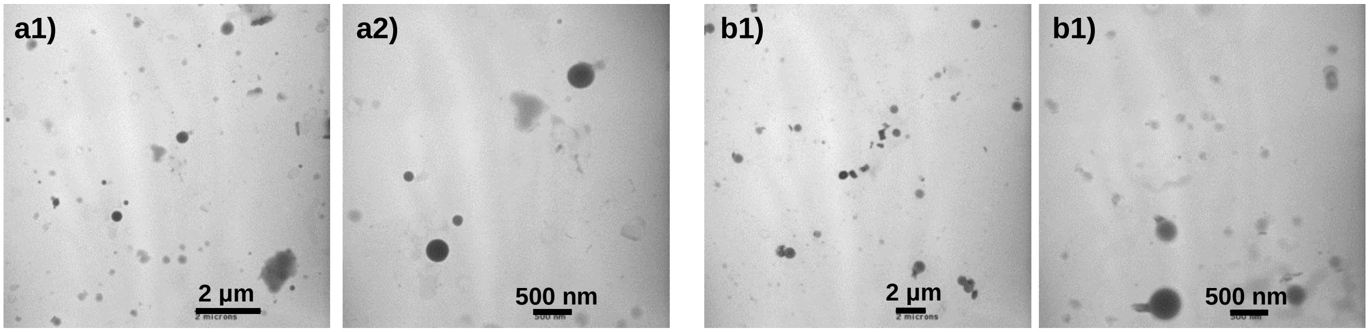

Supplementary Figure 19. TEM images of the self-assembled aggregates of PAA$_{45}$-*b*-PIL$_{23}$ (2 mg/mL THF solution) with the adding of water with water/THF volume ratio (*R)* = 1.6. The adding of water was done within (a) 2 s and (b) 30 min.

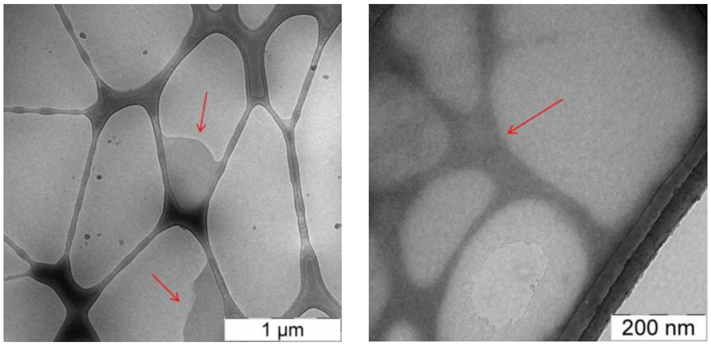

Supplementary Figure 20. Cryo-TEM images of PAA$_{45}$-*b*-PIL$_{23}$. a) After cubosomes were formed at water/THF volume ratio (*R*) = 1.6, additional THF was added to cubosomes dispersion up to *R* = 1.0, leading to coexistence of ill-defined structures and micelles. b) Adding THF to dried cubosomes vanished the cubic structures.

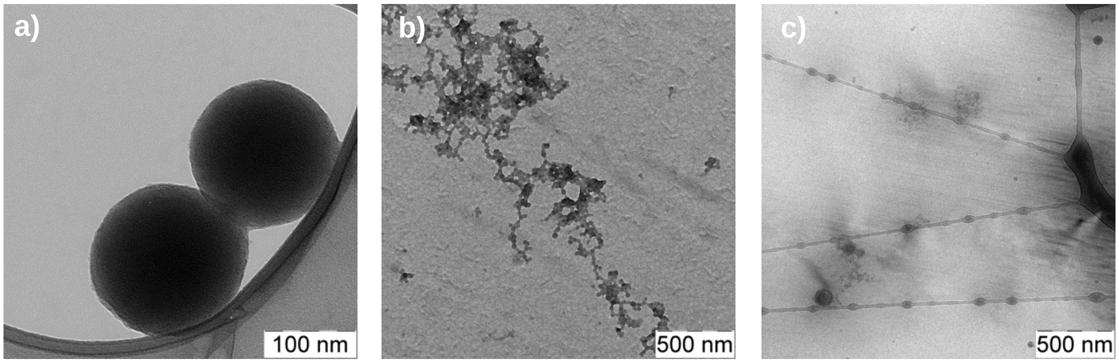

Supplementary Figure 21. TEM images of the self-assembled aggregates of PAA$_{45}$-*b*-PIL$_{23}$ (2 mg/mL DMF solution) with the adding of water at different water/DMF volume ratios (*R*). *R* = 0.9 (a), 1.3 (b), and 1.8 (c).

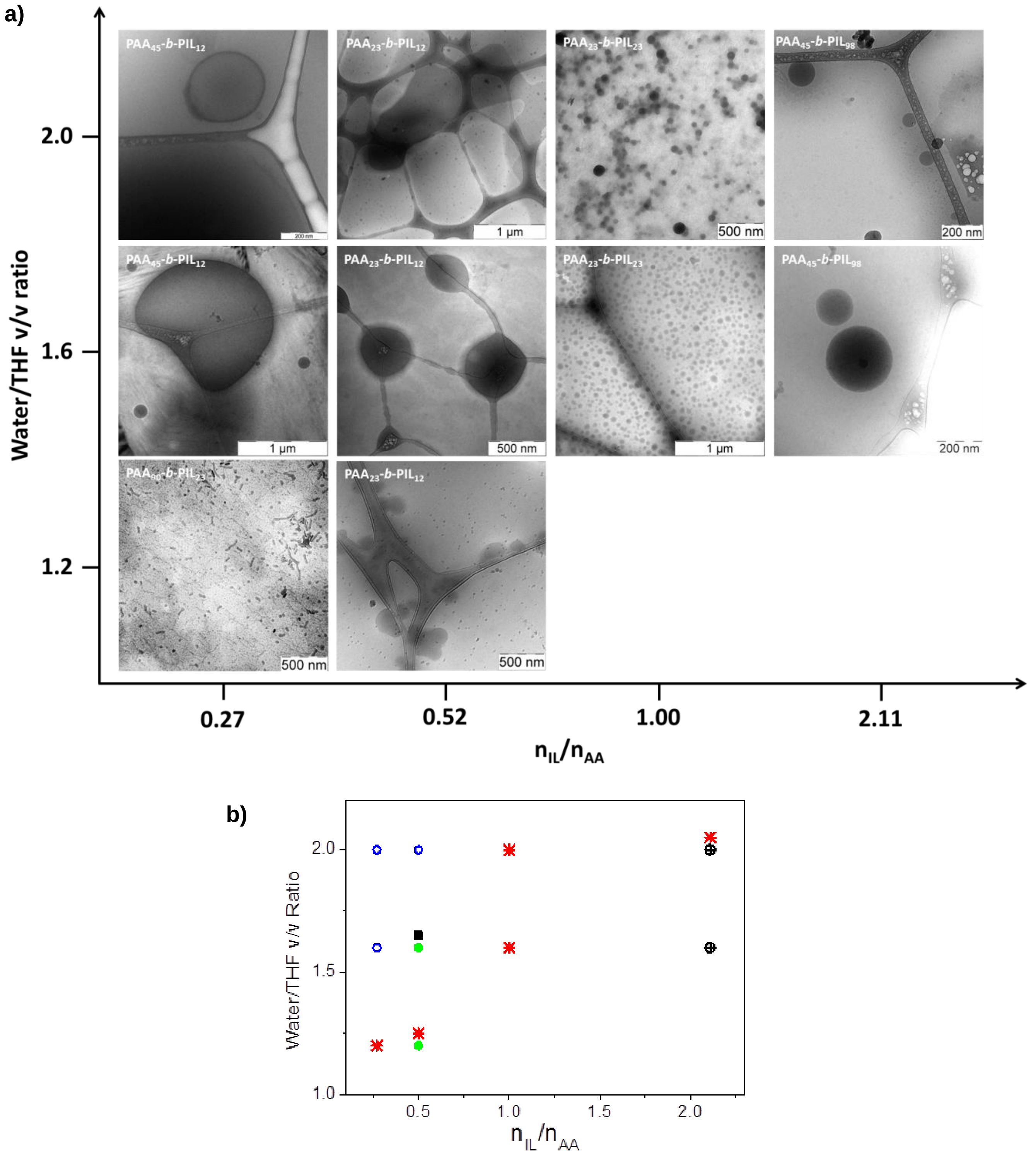

Supplementary Figure 22. a) Cryo-TEM images of the self-assembled aggregates of PAA-b-PILs with different compositions and different water/THF ratio. Self-assembling conditions: 2 mg/mL THF solution, water/THF volume ratio (R) = 1.2-2. n_{IL}/n_{AA} denotes repeating unit ratio of each block. b) Morphology formation of PAA-*b*-PILs in water/THF mixtures as a function of block copolymer

composition and solvent composition. Five types of aggregates were observed – micelles, lamellae (blue), multilamellar vesicles (green), cubic particles (black square), and spherical particles (black circles). The regions with overlapped colors are metastable.

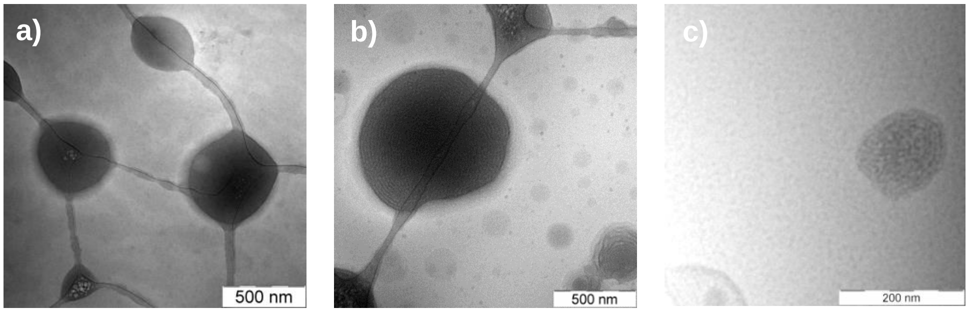

Supplementary Figure 23. Cryo-TEM images of the self-assembled aggregates of PAA_{23}-*b*-PIL_{12} (a), PAA_{45}-*b*-PIL_{23} (b), and PAA_{90}-*b*-PIL_{46} (c) with n_{IL}/n_{AA} = 0.5, but different block contour length Self-assembling conditions: 2 mg/mL THF solution, water/THF volume ratio (*R)* = 1.6.

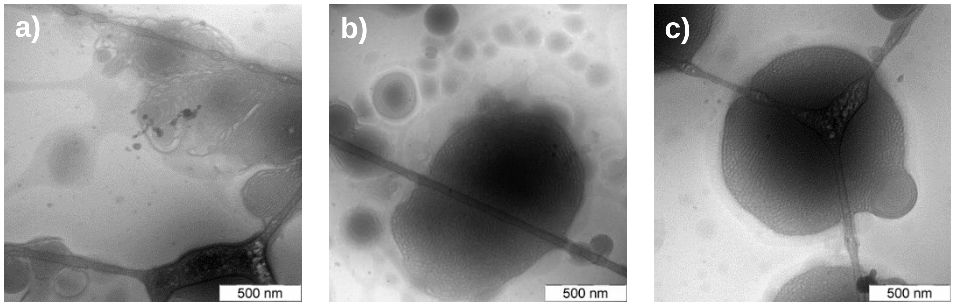

Supplementary Figure 24. CryoTEM images of the self-assembled aggregates of PAA_{45}-*b*-PIL_{23} recorded after a) 1 min, b) 1 hour, and c) 2 days of adding water. Self-assembling conditions: 2 mg/mL THF solution, water/THF volume ratio (*R)* = 1.6.

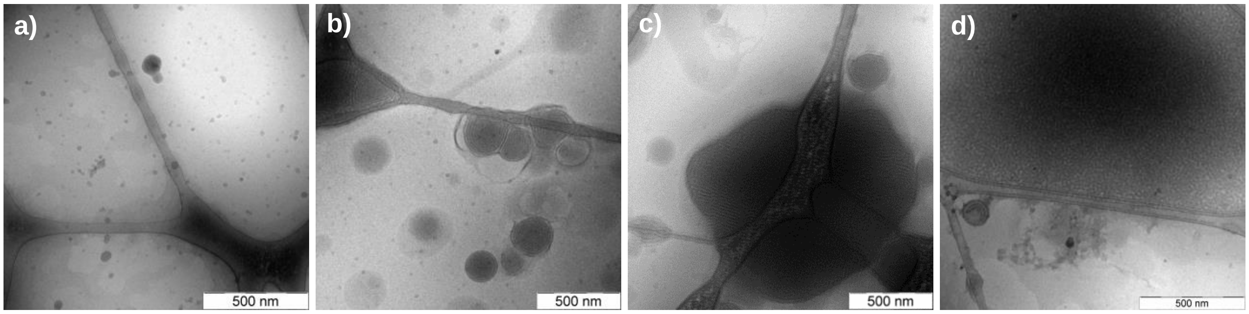

Supplementary Figure 25. Cryo-TEM images of the self-assembled aggregates of PAA_{45}-*b*-PIL_{23}. Self-assembling conditions: 2 mg/mL THF solution of PAA_{45}-*b*-PIL_{23}, water/THF volume ratios *R* = 0.8 (a), 1.2 (b), 1.6 (c), and 2.2 (d).

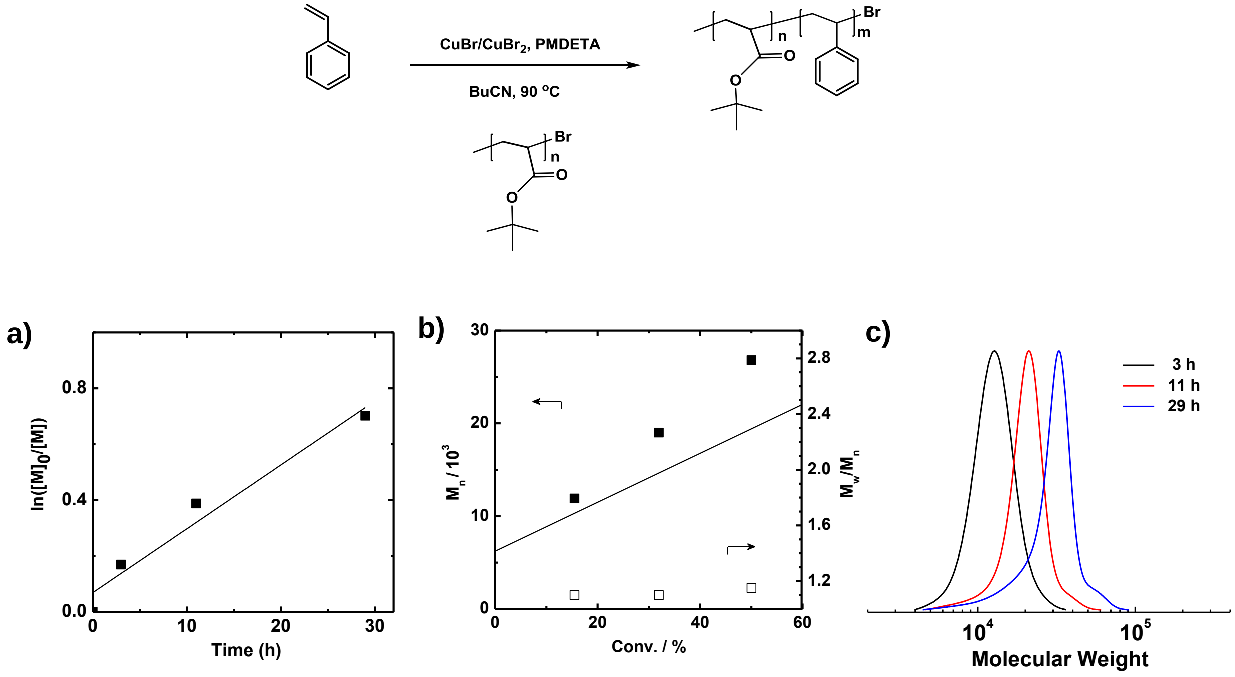

Supplementary Figure 26. Synthesis of P*t*BA$_{45}$-*b*-PS$_{25}$ by ATRP using P*t*BA$_{45}$-Br macroinitiator. Kinetic plot of ln([M]$_0$/[M]) *vs* time (a), plot of M_n and M_w/M_n *vs* conversion calibrated using PS (b), and GPC traces (c) for ATRP of St. Conditions: [St]$_0$/[P*t*BA$_{45}$-Br]$_0$/[CuBr]$_0$/[CuBr$_2$]$_0$/[PMDETA]$_0$ = 50/1/0.95/0.05/1, VBBI$^+$Tf$_2$N$^-$/BuCN = 1/1 (w/w), 90 °C. At 29 h, *conv.* = 50.3%. P*t*BA$_{45}$-*b*-PS$_{25}$: M_n = 26800, PDI = 1.15.

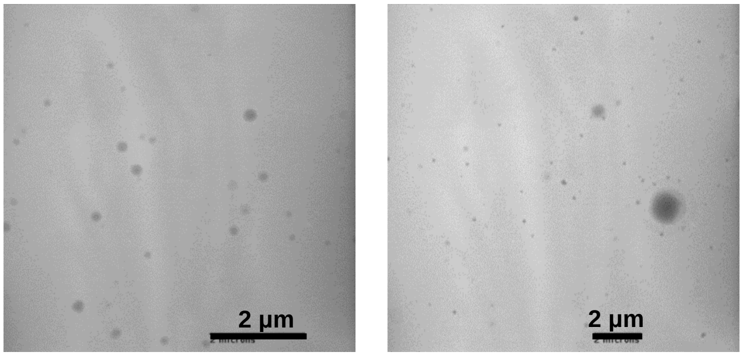

Supplementary Figure 27. TEM images of the self-assembled aggregates of PAA$_{45}$-*b*-PS$_{25}$. Self-assembling conditions: 2 mg/mL THF solution, water/THF volume ratio (*R)* = 1.6.

Supplementary Table 1. GPC and NMR measurements of P*t*BA-*b*-PIL samples.

Entry	Structure	$M_{n,\ theo}$ [a]	$M_{n,\ NMR}$ [b]	$M_{n\ (cal.\ PS)}$ [c]	M_w/M_n [d] (cal. PS)	$M_{n\ (cal.\ PIL)}$ [e]	M_w/M_n [f] (cal. PIL)
1	P*t*BA_{45}-*b*-PIL_{23}	17900	17300	12800	1.12	20600	1.17
2	P*t*BA_{45}-*b*-PIL_{12}	12400	12400	9700	1.09	17700	1.15
3	P*t*BA_{45}-*b*-PIL_{98}	56800	56900	22200	1.13	53100	1.23
4	P*t*BA_{23}-*b*-PIL_{12}	9480	9260	6690	1.10	10500	1.19
5	P*t*BA_{23}-*b*-PIL_{23}	15600	15300	9360	1.09	16800	1.15
6	P*t*BA_{90}-*b*-PIL_{23}	23600	23600	16700	1.07	34300	1.10
7	P*t*BA_{90}-*b*-PIL_{46}	35800	35600	20100	1.10	42300	1.14

[a] The theoretical M_n calculated by the equation of $M_{n,\ calcd} = (conv. \times M_{monomer} \times [M]_0)/[I]_0 + M_{initiator,}$ where *conv.* is the conversion of the IL monomer measured by ^{1}H NMR; $M_{monomer}$ is the molar mass of the IL monomer; $[M]_0$ is the initial concentration of IL monomer; $[I]_0$ is the initial concentration of the macroinitiator P*t*BA-Br; $M_{initiator}$ is the molar mass of the macroinitiator P*t*BA-Br;

[b] The measured $M_{n,\ NMR}$ using the equation of $M_{n,\ NMR} = (A_{NCHN}/A_{tB}) \times 9 \times M_{monomer} + M_{initiator}$, where A_{NCHN} is the integrated area of the NMR peak (~8.7 ppm) of the *H* in NC*H*N of the imidazolium rings, A_{tB} is the integrated area of the NMR peak (~1.5 ppm) of the *H* in the tert-butyl groups, $M_{monomer}$ is the molar mass of the IL monomer, and $M_{initiator}$ is the molar mass of the macroinitiator P*t*BA-Br;

[c] The measured M_n by GPC using polystyrene (PS) as calibration standards;

[d] The measured M_w/M_n by GPC using polystyrene (PS) as calibration standards;

[e] The measured M_n by GPC using polyVBBI$^+$Tf$_2$N$^-_{RAFT}$ [2] as calibration standards;

[f] The measured M_w/M_n by GPC using polyVBBI$^+$Tf$_2$N$^-_{RAFT}$ [2] as calibration standards.

Supplementary References

1. Xia, J. H.; Matyjaszewski, K., Controlled/"living" radical polymerization. Atom transfer radical polymerization catalyzed by copper(I) and picolylamine complexes. *Macromolecules* **1999,** *32* (8), 2434-2437.
2. He, H.; Zhong, M.; Adzima, B.; Luebke, D.; Nulwala, H.; Matyjaszewski, K., A Simple and Universal Gel Permeation Chromatography Technique for Precise Molecular Weight Characterization of Well-Defined Poly(ionic liquid)s. *J. Am. Chem. Soc.* **2013,** *135* (11), 4227-4230.
